# Frequency of heavy vehicle traffic and association with DNA methylation at age 18 years in a subset of the Isle of Wight birth cohort

**DOI:** 10.1093/eep/dvy028

**Published:** 2019-01-23

**Authors:** A Commodore, N Mukherjee, D Chung, E Svendsen, J Vena, J Pearce, J Roberts, S H Arshad, W Karmaus

**Affiliations:** 1 Department of Public Health Sciences, Medical University of South Carolina, Charleston, SC, USA; 2 Division of Epidemiology, Biostatistics, and Environmental Health, University of Memphis, Memphis, TN 38152, USA; 3 Department of Pediatrics, Medical University of South Carolina, Charleston, SC, USA; 4 Faculty of Medicine, University of Southampton, Southampton, UK; 5 The David Hide Asthma and Allergy Research Centre, Isle of Wight, UK

**Keywords:** DNA methylation, air pollution, exposure, traffic, epigenome wide association study

## Abstract

Assessment of changes in DNA methylation (DNA-m) has the potential to identify adverse environmental exposures. To examine DNA-m among a subset of participants (*n* = 369) in the Isle of Wight birth cohort who reported variable near resident traffic frequencies. We used self-reported frequencies of heavy vehicles passing by the homes of study subjects as a proxy measure for TRAP, which were: never, seldom, 10 per day, 1–9 per hour and >10 per hour. Methylation of cytosine-phosphate-guanine (CpG) dinucleotide sequences in the DNA was assessed from blood samples collected at age 18 years (*n* = 369) in the F1 generation. We conducted an epigenome wide association study to examine CpGs related to the frequency of heavy vehicles passing by subjects’ homes, and employed multiple linear regression models to assess potential associations. We repeated some of these analysis in the F2 generation (*n* = 140). Thirty-five CpG sites were associated with heavy vehicular traffic. After adjusting for confounders, we found 23 CpGs that were more methylated, and 11 CpGs that were less methylated with increasing heavy vehicular traffic frequency among all subjects. In the F2 generation, 2 of 31 CpGs were associated with traffic frequencies and the direction of the effect was the same as in the F1 subset while differential methylation of 7 of 31 CpG sites correlated with gene expression. Our findings reveal differences in DNA-m in participants who reported higher heavy vehicular traffic frequencies when compared to participants who reported lower frequencies.

## Introduction

Evidence for the health impacts of air pollution has been mounting up for several decades [1–3]. Exposure to ambient air pollutants is associated with both acute and chronic health effects and the impacts are felt on global and local scales [4]. Interestingly, the observed adverse health effects are seen even at very low levels of air pollution exposure, and it is unclear whether any threshold exists (i.e. a concentration below which there are no effects on health) [5]. The concentration of air pollutants can differ in a small geographic area depending on local ambient conditions [6]. Key environmental factors that significantly affect local air quality includes proximity to traffic, wood burning, coal burning, dry cleaning, motor vehicle exhaust and industrial emissions, among others [7–14]. Exposures to such environmental factors are associated with asthma exacerbation [15], although their contribution to the development of the disease is uncertain [16].

For an environmental factor such as traffic, it is often necessary to investigate simple proxies such as distance to roadways and traffic estimates or counts, to help assign individual exposures and account for spatial variability. For instance, there is increasing evidence that living near heavy traffic is associated with increased rates of asthma, cardiovascular disease and dementia [17–21], and chronic air pollution exposure gradients at such small scales are associated with adverse cardiorespiratory effects [22]. In the absence of neighborhood level, air pollution measurements, proximity to traffic, traffic volume, among other methods, can be employed [6, 20, 23–30]. Such substitutes facilitate the characterization of smaller-scale air pollution exposures, and have been operative in some health studies [31–33].

Recent evidence indicates that epigenetics may play an important role in mediating the health effects of air pollution [34]. Indeed, it has been suggested that the extent of epigenetic markers can change progressively and help construct cumulative exposure patterns over time [35]. Interestingly, changes in epigenetic markers can result from exposure to a risk factor such as air pollution, and such changes can potentially serve as predictive biomarkers of susceptibility to adverse health [36]. The epigenetic marker of DNA methylation (DNA-m), which is the addition of a methyl group to cysteine in cytosine-phosphate-guanine (CpG) dinucleotides sequences in the DNA, is reported to be related to air pollution exposures [37, 38], and adverse respiratory health [39], including asthma [40, 41].

Changes in the epigenome and gene expression may be induced by exposure to air pollution [34, 42] and this is relevant to the development of several pathophysiological processes. Difflerential blood DNA-m in response to air pollution exposure from sources such as traffic has been reported [43–45]. We cannot or rarely can directly assess DNA-m in target tissues, such as the lung. However, for many biomarkers, blood changes are considered to constitute a window through which specific processes in other tissues can be assessed. In addition, during development, blood and airways stem from the mesoderm and may represent to have a similar development and susceptibility [46]. For these reasons, the effects of TRAP on epigenome in blood samples represent informative biomarkers of change in the airways.

Given that (i) TRAP exerts its greatest impact on local scales, particularly near roadways [47] and (ii) the mechanistic basis for the effects of TRAP on the epigenome is not well delineated [48], additional studies can provide further evidence and advance the current state of the science [49]. Accordingly, we used the self-reported frequencies of heavy vehicles passing by the homes of study subjects as a proxy measure for TRAP and evaluated their associations with the methylation of CpG sites among 18-year-old participants in the Isle of Wight (IoW) birth cohort, UK (*n* = 369). Our motivating questions were:

1. Which specific CpG sites are associated with heavy vehicular traffic in the birth cohort?
2. Are there any trends in the association between differential DNA-m (both higher and lower) and the frequency of exposure to heavy vehicular traffic?

## Results

### Characteristics of Study Population

Eighteen percent of the subjects (*n* = 67) reported never having any heavy vehicles passing by their homes while 82% reported some heavy vehicular traffic outside their homes (Table 1). About 20% had a history of maternal smoking and nearly 50% were exposed to tobacco smoke outside their homes and before age 4 (Table 1). About a quarter of the subjects were current smokers who started smoking at an average of 14.5 (SD 1.5) years. A vast majority of the subjects present a middle class status (72%) with over 90% still living at home with their parents and 70% living in a private residential property. The average body mass index (BMI) was 23.6 (SD 4.3). In this subset with DNA-m, there were more females than males (66% vs. 34%) due to the study design (following until pregnancy) (Table 1).

**Table 1:** Comparison of population characteristics of participants in the whole cohort and those with DNA-m at age 18 years

	Whole cohort (*n* = 1313)	Sample with DNA-m (*n* = 369)	*P*-value
	*n* (%)	*n* (%)	
**Gender**			<0.0001
Female	660 (50.27)	245 (66.4)	
Male	653 (49.73)	124 (33.6)	
**Maternal smoking status**			0.24
No	1002 (76.31	292 (79.1)	
Yes	305 (23.23)	75 (20.3)	
Unanswered	6 (0.46)	2 (0.5)	
**Frequency of heavy vehicles passing by home**			0.783
>10 per hour	274 (20.87)	77 (20.9)	
1–9 per hour	223 (16.98)	69 (18.7)	
10 per day	119 ( 9.06)	36 (9.8)	
Seldom	427 (32.52)	120 (32.5)	
Never	241 (18.35)	67 (18.2)	
Missing	29 (2.21)		
**Exposure to smoking outside home**			0.41
Yes	598 (45.54)	180 (48.8)	
No	663 (50.50)	181 (49.1)	
Unanswered	52 (3.96)	8 (2.2)	
**Current smoking status**			0.3
No	910 (69.31)	270 (73.2)	
Yes	368 (28.03)	95 (25.8)	
Unanswered	35 (2.67)	4 (1.1)	
**Any exposure to environmental tobacco smoke (at 10 years)**			0.23
No	716 (54.5)	223 ( 60.4)	
Yes	492 (37.5)	132 (35.8)	
Unanswered	105 (8.0)	14 (3.8)	
**Any exposure to environmental tobacco smoke (at birth, 1 year, 2 year or 4 years)**			0.11
No	602 (45.8)	186 (50.4)	
Yes	708 (53.9)	182 (49.3)	
Unanswered	3 (0.23)	1 (0.27)	
**Socio-economic status**			0.73
High	103 (7.84)	33 ( 8.9)	
Mid	952 (72.51)	267 (72.4)	
Low	177 (13.48)	58 (15.7)	
Unanswered	81 (6.17)	11 (2.9)	
**Living with parents**			0.11
Yes	1158 (88.19)	342 (92.7)	
No	129 (9.82)	27 (7.3)	
Missing	26 (1.98)		
**Type of residential property**			0.78
Rented privately	145 (11.0)	28 (7.5)	
Rented council/housing association	209 (15.9)	65 (17.6)	
Owned privately	908 (69.1)	271 (73.4)	
Other	18 (1.37)	5 (1.4)	
Missing	33 (2.51)		
Median (p5, p95)			
**BMI**	22.1 (18.2, 32.1)	22.5 (18.7, 32.1)	0.09
**Age subject started smoking**	15 (12, 17)	15 (12, 17)	0.88
**Time living in present house**	48 (6, 48)	48 (6, 48)	0.48

Percentage has been rounded up to whole numbers, where applicable.

### Which Specific CpG Sites Are Associated with Heavy Vehicular Traffic in the IoW Cohort?

There were a total of 371 CpG sites that were associated with heavy vehicular traffic frequency based on *ttscreening* results. However, we chose the top CpGs with a cutoff percentage of 70 [*m* = 70 across 100 total iterations (*i *=* *100)] was used to determine the final pool of potentially important CpG sites (in our case 35 of 371 had a cutoff percentage between 70 and 94). Therefore, a final group of 35 CpGs was selected in step 1 (Tables 2 and 3). The 35 CpG sites are listed in the order of significance based on the epigenome-wide association analysis results. Over 30% of these CpGs were located on Chromosome 1. The identified CpG sites were associated with 34 different genes (two CpG sites—cg11156891 and cg12407057—mapped to one gene *ANKRD65*). A majority of the CpG sites were located in the body of the identified gene (24 of 35); 4 were 200–1500 bases upstream of the transcriptional start site (TSS), while 2 were 0–200 of the TSS; 3 were within the 5′ untranslated region, and 2 were over 50 kb from the nearest gene (Table 3).

**Table 2:** Summary of (a) CpG sites found this is exploratory study, (b) genes associated with the CpGs and (c) list of chemicals documented in the comparative toxicogenomics database that are related to air pollution

(a) CpG site	(b) Identified gene in this study	(c) Comparative toxicogenomics database chemical (related to air pollution)
**cg25895913**	CDH4	Benzo(a)pyrene, 7,8-dihydro-7,8-dihydroxybenzo(a)pyrene 9,10-oxide
**cg11156891**	ANKRD65	Benzo(a)pyrene
**cg12407057**	ANKRD65	Benzo(a)pyrene
**cg20747739**	FAM132A	Benzo(a)pyrene
**cg18565510**	ACAP3	Benzo(a)pyrene
**cg24843003**	DAZAP1	Benzo(a)pyrene, air pollutants, occupational, 1-hydroxypyrene
**cg15730464**	LGI2	Benzo(a)pyrene, soot, tobacco smoke pollution
**cg16196077**	RTKN2	Benzo(a)pyrene, ozone
**cg02707264**	MYRIP	Benzo(a)pyrene
**cg03476673**	CRISPLD2	7,8-Dihydro-7,8-dihydroxybenzo(a)pyrene 9,10-oxide
**cg07023532**	ACOT4	Benzo(a)pyrene
**cg20255272**	VWA1	None
**cg12417992**	SLC6A9	Benzo(a)pyrene, ozone, dibenzo(a, l)pyrene
**cg04154465**	WNT2B	None
**cg12813768**	SYCP1	None
**cg14162906**	TMEM222	7,8-Dihydro-7,8-dihydroxybenzo(a)pyrene 9,10-oxide
**cg24361098**	BCL11A	Benzo(a)pyrene
**cg16147794**	SLC16A10	Benzo(a)pyrene
**cg16668397**	JPH3	None
**cg26419883**	TRPM5	Benzo(a)pyrene
**cg21775675**	TMEM161B	None
**cg04794690**	PADI3	Benzo(a)pyrene, particulate matter
**cg06942649**	FBXO25	Benzo(a)pyrene
**cg18459806**	NIN	Benzo(a)pyrene, 7,8-dihydro-7,8-dihydroxybenzo(a)pyrene 9,10-oxide
**cg20631351**	PALM	Smoke
**cg00347824**	NSMAF	Benzo(a)pyrene
**cg17053854**	SEPT9	Benzo(a)pyrene, 7,8-dihydro-7,8-dihydroxybenzo(a)pyrene 9,10-oxide
**cg25324786**	RASA3	Benzo(a)pyrene, 7,8-dihydro-7,8-dihydroxybenzo(a)pyrene 9,10-oxide
**cg26720961**	TSNARE1	None
**cg05575058**	FAM164A	Benzo(a)pyrene
**cg15742605**	SAMD11	Smoke
**cg26185508**	CDCP2	None
**cg02378006**	UNC5B	None
**cg08462127**	MYOM2	Benzo(a)pyrene
**cg11017318**	SYT16	None

This information was obtained from the UCSC GenomeBroswer (GRCh37/hg19).

**Table 3:** Descriptive statistics of the DNA-m of the significant CpG sites in whole blood samples at age 18 (*n* = 369)

Raw *P*-values from *tt screening*	Chromosome (hg19)	CpG site	Location	Coordinate (hg19)	Minimum	5th Pctl	Median	95th Pctl	Maximum
3.69E–06	1	cg25895913	Body	54619445	–0.804	–0.414	0.025	0.366	0.612
2.17E–06	1	cg11156891	Body	1373678	–1.485	–0.881	–0.125	1.191	2.589
6.84E–06	1	cg12407057	Body	44500834	–0.971	–0.517	–0.087	0.900	2.540
1.62E–06	17	cg20747739	TSS1500	75463180	–0.812	–0.358	0.004	0.335	0.709
8.53E–06	14	cg18565510	Body	51293793	–0.897	–0.500	–0.002	0.464	0.844
2.22E–05	8	cg24843003	Body	79578035	–0.904	–0.449	0.003	0.416	1.093
2.21E–05	2	cg15730464	Body	60748951	–1.254	–0.655	0.016	0.562	0.825
1.48E–05	11	cg16196077	TSS200	2434192	–1.311	–0.798	–0.048	0.880	1.512
7.14E–05	1	cg02707264	5′UTR	1366206	–0.744	–0.365	0.005	0.321	0.634
3.00E–05	1	cg03476673	5′UTR	1183257	–1.149	–0.598	–0.015	0.608	1.208
2.33E–04	3	cg07023532	TSS1500	39851931	–0.968	–0.505	–0.013	0.488	1.229
7.50E–05	19	cg20255272	Body	711001	–1.700	–1.046	0.104	0.460	0.807
5.26E–05	1	cg12417992	Body	113045271	–0.572	–0.347	–0.025	0.396	0.716
8.95E–05	1	cg04154465	Body	1238702	–1.351	–0.588	0.004	0.600	1.390
5.58E–05	1	cg12813768	Body	115398123	–1.643	–0.861	0.002	0.885	1.441
9.81E–05	1	cg14162906	TSS1500	27647606	–0.713	–0.392	–0.004	0.379	0.803
1.22E–04	8	cg24361098	Body	143386260	–1.080	–0.634	0.015	0.537	0.996
3.50E–05	16	cg16147794	Body	87720712	–1.217	–0.578	–0.022	0.660	1.565
1.20E–04	5	cg16668397	Body	87441487	–0.565	–0.386	–0.008	0.462	0.785
1.16E–04	8	cg26419883	Body	2017365	–0.818	–0.419	–0.007	0.418	0.702
1.52E–04	13	cg21775675	∼50 kb upstream of TMEM161B	114866221	–0.987	–0.460	–0.005	0.518	1.296
1.42E–04	19	cg04794690	Body	1409547	–1.217	–0.500	0.002	0.525	0.719
7.64E–05	10	cg06942649	Body	64028521	–1.588	–0.948	0.016	0.757	1.066
2.30E–04	8	cg18459806	5′UTR	436813	–0.610	–0.359	0.002	0.373	0.807
2.18E–04	8	cg20631351	Body	59571961	–0.679	–0.379	–0.005	0.362	0.818
2.01E–04	20	cg00347824	Body	60460465	–1.267	–0.538	0.028	0.458	1.166
4.21E–05	1	cg17053854	Body	17768059	–0.474	–0.289	0.001	0.277	0.682
2.55E–04	1	cg25324786	Body	879383	–0.935	–0.568	0.021	0.466	0.985
3.05E–04	14	cg26720961	Body	62279992	–1.221	–0.556	–0.009	0.583	1.659
4.63E–04	4	cg05575058	TSS1500	25087441	–0.907	–0.455	–0.029	0.459	0.804
3.39E–04	6	cg15742605	Body	111405660	–0.645	–0.417	–0.023	0.531	1.064
2.17E–04	10	cg26185508	TSS200	73026288	–0.909	–0.493	–0.007	0.487	0.948
1.12E–04	1	cg02378006	Body	1366274	–1.212	–0.576	–0.015	0.595	1.240
2.69E–04	16	cg08462127	Body	84870203	–0.832	–0.434	0.004	0.431	0.724
5.65E–04	14	cg11017318	∼200 kb upstream of SYT16	74057654	–0.607	–0.382	0.004	0.369	0.772

We also assessed answers to other traffic-related questions such as ‘***How often do cars pass your house or on the street less than 100 meters away?***’ and ‘***How frequently are you annoyed by outdoor air pollution (from traffic industry, etc) in your home if you keep the window open?***’. However, these did not have much variability nor did they yield any significant results with the *ttScreening* package. The CpG by CpG analysis also did not show any statistically significant results for Any versus Never reports of heavy vehicular traffic frequency after adjusting for false discovery rate (FDR; all adjusted *P*-values were ≥0.4). There was no association between heavy vehicular traffic frequency and cg05575921, located in the aryl hydrocarbon receptor repressor (*AHRR*) gene. However, there appeared to be an association with self-reported smoking status (among current smokers), tobacco smoke exposure assessed through a questionnaire administered at 10 years and environmental tobacco smoke exposure (Table 4).

**Table 4:** Results for linear models for methylation of cg07555921 (*AHRR*), considered to be a marker for smoking

Parameter	Estimate	Standard error	*P*-value
**Model 1: Exposure variable only: frequency of heavy vehicular traffic (ref=never), *n*** **=** **369**			
Heavy vehicular traffic frequency: >10/hour	–0.10	0.11	0.4
Heavy vehicular traffic frequency: 1–9/hour	0.13	0.11	0.2
Heavy vehicular traffic frequency: 10/day	–0.05	0.13	0.7
Heavy vehicular traffic frequency: Seldom	0.08	0.10	0.4
**Model 2: Smoking related variables only, *n*** **=** **342**			
Maternal smoking	–0.07	0.09	0.4
Current smoking status	0.77	0.08	**<.0001**
Smoking outside the home	0.09	0.07	0.2
Any environmental tobacco smoke exposure	–0.16	0.08	***0.05***
Tobacco smoke exposure at 0–4 years	0.09	0.06	0.2
Tobacco smoke exposure at 10 years	0.15	0.09	***0.09***
**Model 3: All variables considered apriori among all subjects, *n*** **=** **329**			
Heavy vehicular traffic frequency: >10/hour	–0.14	0.10	0.2
Heavy vehicular traffic frequency: 1–9/hour	–0.04	0.10	0.7
Heavy vehicular traffic frequency: 10/day	–0.08	0.13	0.5
Heavy vehicular traffic frequency: Seldom	–0.04	0.09	0.7
Maternal smoking	–0.10	0.10	0.3
Current smoking status	0.75	0.08	**<.0001**
Smoking outside the home	0.11	0.07	0.1
Any environmental tobacco smoke exposure	–0.18	0.09	**0.04**
Tobacco smoke exposure at 0–4 years	0.09	0.07	0.2
Tobacco smoke exposure at 10 years	0.17	0.09	***0.06***
High SES (ref = low SES)	–0.22	0.13	0.1
Mid SES (ref = low SES)	–0.04	0.09	0.6
BMI	0.02	0.01	**0.007**
Gender	0.04	0.07	0.5
**Model 4: All variables considered apriori among current smokers, *n*** **=** **77**			
Heavy vehicular traffic frequency: >10/hour	–0.13	0.30	0.7
Heavy vehicular traffic frequency: 1–9/hour	0.48	0.34	0.2
Heavy vehicular traffic frequency: 10/day	0.41	0.37	0.3
Heavy vehicular traffic frequency: Seldom	0.20	0.31	0.5
Maternal smoking	–0.07	0.26	0.8
Age subject started smoking	0.20	0.06	**0.002**
Smoking outside the home	0.79	0.32	**0.02**
Any environmental tobacco smoke exposure	–0.33	0.23	0.2
Tobacco smoke exposure at 0–4 years	0.14	0.20	0.5
Tobacco smoke exposure at 10 years	0.32	0.25	0.2
High SES (ref = low SES)	–0.68	0.44	0.1
Mid SES (ref = low SES)	–0.04	0.24	0.9
BMI	0.03	0.02	0.1
Gender	0.04	0.21	0.9

*P*-values in bold and bold italics denote statistical significance less than or equal to 0.05 and 0.1 respectively.

### Gene Set Enrichment Analysis

Using the bioinformatic resource ToppGene Suite [50], we performed a gene enrichment analysis to determine the pathway(s) associated with genes of the significant CpG sites [the respective genes that had the exact CpG coordinates, or if the CpG was located between two genes (i.e. intergenic CpGs), we selected the gene with the closest proximity to the intergenic CpG]. Input parameters for the gene enrichment analysis were as follows: All 34 genes were included in the training set, the hypergeometric probability mass function was used to calculate *P*-values, and the FDR was controlled at 0.05 using the Benjamini–Hochberg method.

**Figure 1:**
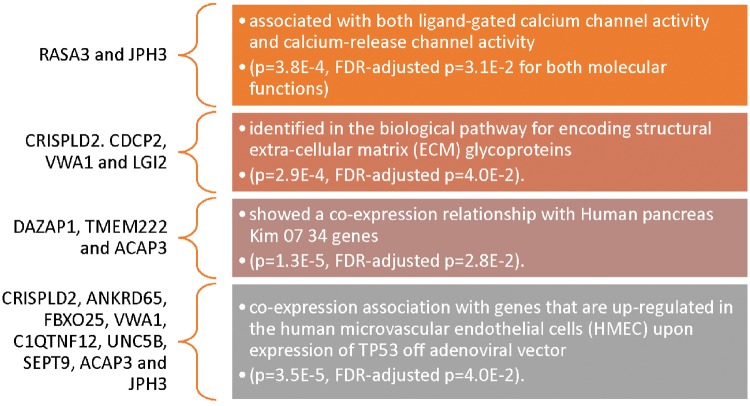
Results of gene enrichment analysis identifying potential pathway(s) associated with the genes identified with the significant CpG sites in this study. More details on the gene enrichment analysis can be found in the supplementary material provided

Two genes, *RASA3* and *JPH3*, were associated with both ligand-gated calcium channel activity and calcium-release channel activity (Fig. 1). Four genes (*CRISPLD2*, *CDCP2*, *VWA1* and *LGI2*) were identified in the biological pathway for encoding structural extra-cellular matrix (ECM) glycoproteins (all FDR-adjusted *P* < 0.05, Fig. 1). Additionally, a total of nine genes (*CRISPLD2*, *ANKRD65*, *FBXO25*, *VWA1*, *C1QTNF12*, *UNC5B*, *SEPT9*, *ACAP3* and *JPH3*) came up as having a coexpression association with genes that are upregulated in the human microvascular endothelial cells.

When the analysis is separated into the 23 more methylated and 11 less methylated CpG sites, the latter is identified in the following gene families: zinc fingers (*P* = 5.3E–3, FDR-adjusted *P* = 1.7E–2), synaptotagmins (*P* = 5.6E–3, FDR-adjusted *P* = 1.7E–2) and A-kinase anchoring proteins (*P* = 9.9E–3, FDR-adjusted *P* = 2.0E–2) but no molecular functions are identified. On the other hand, the former is associated with five different molecular functions:

- calcium-release channel activity (*P* = 1.7E–4, FDR-adjusted *P* = 1.2E–2),
- ligand-gated calcium channel activity (*P* = 1.7E–4, FDR-adjusted *P* = 1.2E–2),
- intracellular ligand-gated ion channel activity (*P* = 6.E–4, FDR-adjusted *P* = 2.9E–2),
- succinyl-CoA hydrolase activity (*P* = 1.2E–3, FDR-adjusted *P* = 4.3E–2) and
- metal ion transmembrane transporter activity (*P* = 1.7E–3, FDR-adjusted *P* = 4.7E–2).

Additionally, 17 gene families are identified with these 23 more methylated CpGs including CD molecules Type I classical cadherins and Peptidyl arginine deiminases (both: *P* = 5.2E–3, FDR-adjusted *P* = 3.4E–2).

### Association with Air Pollutants in the Comparative Toxicogenomics Database

The analysis did not reveal any links to air pollutants, as there is currently not enough data on air pollution to factor into biological pathway analyses. However, a search of the description and page index of each gene provided information on reported chemicals related to air pollution in the comparative toxicogenomics database [51]. All but three genes were associated with chemical(s) found in air pollution, e.g. ‘benzo(a)pyrene’, ‘7,8-dihydro-7,8-dihydroxybenzo(a)pyrene 9,10-oxide’, ‘smoke’ and even ‘particulate matter’ (Table 2).

### Diseases for Which the Identified Genes Are Enriched

The gene enrichment analysis also identified the following diseases that associated with six of the genes identified with the significant CpG sites in this study

- *SEPT9* is associated with Orbital separation diminished, hereditary neuralgic amyotrophy (HNA), Brachial Plexus Neuritis, Epiphyses, hemoglobinopathies.
- *NIN* is associated with Orbital separation diminished, Seckel syndrome, HNA, Spondyloepimetaphyseal dysplasia with multiple dislocations, Lumbar scoliosis.
- *JPH3* is associated with Huntington disease-like 2, Akinetic rigid syndrome, Brachial Plexus Neuropathies
- *MYOM2* is associated with IgA myeloma, Osteosclerotic Myeloma
- *BCL11A* is associated with Amyotrophy, HNA, hemoglobinopathies, F-cell distribution, fetal hemoglobin levels
- *PADI3* is associated with Uncombable hair, generalized trichodysplasia

### Are There Any Trends in the Association between the Frequencies of Heavy Vehicular Traffic on the IoW and DNA-m?

An initial ANOVA revealed a total of 24 of 35 CpGs with significantly different DNA-m (*P* < 0.05) when those who reported no heavy vehicles (never) were compared with those who reported any heavy vehicular traffic (Table 5). Further evaluations (using never, seldom, 10 per day, 1–9 per hour or >10 per hour levels) showed all 35 CpGs had significantly different DNA-m for at least one of the five categories of heavy vehicle traffic *P* ≤ 0.01 (Table 5).

**Table 5:** Analysis of variance for unadjusted DNA-M of CpG sites in whole blood samples at age 18 (*n* = 369)

CpG site	Any (*n* = 302) versus no (*n* = 67) heavy vehicle traffic frequency			Five categories of heavy vehicle traffic frequency: (1) never (*n* = 67), (2) seldom (*n* = 120), (3) 10/day (*n* = 36), (4) 1–9/hour (*n* = 69), (5) >10/hour (*n* = 77)		
	Mean square	*F* value (df = 1)	*P* value	Mean square	*F* value (df = 4)	*P* value
cg25895913	0.53	9.4	0.002	0.33	6.1	<0.0001
cg11156891	4.55	9.7	0.002	2.61	5.7	0.0002
cg12407057	4.55	9.7	0.002	1.37	5.2	0.0004
cg20747739	0.30	6.6	0.01	0.27	6.2	<0.0001
cg18565510	0.84	9.7	0.002	0.44	5.2	0.0005
cg24843003	0.97	12.3	0.0005	0.40	5.1	0.0005
cg15730464	1.59	13.0	0.0004	0.78	6.6	<0.0001
cg16196077	1.32	5.0	0.03	1.39	5.5	0.0003
cg02707264	0.06	1.3	**0.3**	0.22	5.1	0.0006
cg03476673	1.38	9.5	0.002	0.71	5.0	0.0007
cg07023532	1.62	17.6	<.0001	0.51	5.6	0.0002
cg20255272	0.66	3.6	**0.06**	0.73	4.1	0.003
cg12417992	0.37	8.2	0.0046	0.20	4.5	0.001
cg04154465	0.48	3.4	**0.07**	0.56	4.0	0.003
cg12813768	1.08	4.3	0.04	1.13	4.7	0.001
cg14162906	0.49	8.9	0.003	0.26	4.7	0.001
cg24361098	1.38	11.1	0.0009	0.56	4.6	0.001
cg16147794	0.27	1.8	**0.2**	0.81	5.6	0.0002
cg16668397	0.42	6.8	0.01	0.24	3.9	0.004
cg26419883	0.25	4.1	**0.05**	0.26	4.4	0.002
cg21775675	0.33	3.7	**0.06**	0.36	4.1	0.003
cg04794690	1.31	14.8	0.0001	0.49	5.5	0.0003
cg06942649	2.04	8.2	0.004	1.06	4.3	0.002
cg18459806	0.29	5.7	0.02	0.20	4.1	0.003
cg20631351	0.29	5.5	0.02	0.18	3.5	0.008
cg00347824	0.62	6.4	0.01	0.37	3.9	0.004
cg17053854	0.18	5.9	0.02	0.13	4.6	0.001
cg25324786	0.15	1.6	**0.2**	0.36	4.0	0.004
cg26720961	0.01	0.1	**0.7**	0.61	5.0	0.0006
cg05575058	0.64	8.6	0.004	0.27	3.7	0.006
cg15742605	0.21	2.5	**0.1**	0.28	3.5	0.008
cg26185508	0.82	9.4	0.002	0.39	4.5	0.002
cg02378006	0.39	3.1	**0.08**	0.54	4.5	0.001
cg08462127	0.15	2.2	**0.1**	0.30	4.4	0.002
cg11017318	0.28	5.3	0.02	0.18	3.5	0.008

*P*-values in bold and bold italics denote statistical significance less than or equal to 0.05 and 0.1 respectively.

After adjusting for history of maternal smoking, environmental tobacco smoke exposure (0–4 years and/or at 10 years), SES, gender, BMI, current smoking status and/or exposure to smoke outside the home, 34 CpGs remained statistically significant depending on the category of heavy vehicle traffic frequency reported (*P* ≤ 0.05, range for *n* = 329–369) (Table 6). We also present results for linear models for the top 35 CpG sites identified with the *ttscreening* method after adjusting for all confounding factors considered a priori in this study, and the results are similar to Table 6 where associations are still present in 34 of 35 CpG sites for at least one category of the exposure variable (Supplementary Table S1).

**Table 6:** Results for multiple linear regression models for CpG sites associated with the frequency of heavy vehicles passing by all subjects’ homes

CpG	Associated gene	Heavy vehicle frequency (ref = never)	Estimate	Standard error	*P* value	Significant covariates in final model	Dunnett's test (LSMEAN = never)	Linear trend test (*F* value, df = 1) *P value*	Direction of methylation
cg25895913 (*n* = 355)	CDH4	>10/hour	0.15	0.04	0.0002	Tobacco smoke exposure (at 10 years); gender	***	18.16	**↑**
		1–9/hour	0.17	0.04	<.0001		***	*<0.0001*	
		10/day	0.06	0.05	0.2				
		Seldom	0.05	0.04	0.2				
cg11156891 (*n* = 329)	ANKRD65	>10/hour	–0.48	0.13	0.0002	Maternal smoking; tobacco smoke exposure (0–4 years and at 10 years); SES; gender; BMI; current smoking status; exposure to smoke outside the home	***	14.49	**↓**
		1–9/hour	–0.29	0.13	0.03		*	*0.0002*	
		10/day	–0.19	0.15	0.2				
		Seldom	–0.12	0.11	0.3				
cg12407057 (*n* = 329)	ANKRD65	>10/hour	–0.33	0.10	0.0006	Maternal smoking; tobacco smoke exposure (0–4 years and at 10 years); SES; gender; BMI; current smoking status; exposure to smoke outside the home	***	15.51	**↓**
		1–9/hour	–0.24	0.10	0.01		*	*0.0001*	
		10/day	–0.08	0.12	0.5				
		Seldom	–0.07	0.09	0.4				
cg20747739 (*n* = 362)	FAM132A	>10/hour	0.14	0.04	<.0001	Gender; BMI	***	19.7	**↑**
		1–9/hour	0.11	0.04	0.002		**	*<0.0001*	
		10/day	0.04	0.04	0.3				
		Seldom	0.03	0.03	0.4				
cg18565510 (*n* = 362)	ACAP3	>10/hour	0.20	0.05	<.0001	Gender; BMI	***	15.33	**↑**
		1–9/hour	0.14	0.05	0.008		**	*0.0001*	
		10/day	0.09	0.06	0.1				
		Seldom	0.07	0.05	0.1				
cg24843003 (*n* = 369)	DAZAP1	>10/hour	0.19	0.05	<.0001	Gender	***	14.73	**↑**
		1–9/hour	0.15	0.05	0.002		**	*0.0001*	
		10/day	0.07	0.06	0.2				
		Seldom	0.09	0.04	0.03		*		
cg15730464 (*n* = 348)	LGI2	>10/hour	0.28	0.06	<.0001	Tobacco smoke exposure ( at 10 years); SES; gender	***	20.56	**↓**
		1–9/hour	0.17	0.06	0.006		**	*<0.0001*	
		10/day	0.03	0.07	0.7				
		Seldom	0.16	0.05	0.004		**		
cg16196077 (*n* = 336)	RTKN2	>10/hour	–0.27	0.09	0.003	Maternal smoking; tobacco smoke exposure (0–4 years and at 10 years); SES; gender; current smoking status; exposure to smoke outside the home	**	12.62	**↓**
		1–9/hour	–0.25	0.09	0.007		**	*0.0004*	
		10/day	–0.14	0.11	0.2				
		Seldom	0.02	0.08	0.8				
cg02707264 (*n* = 329)	MYRIP	>10/hour	–0.11	0.04	0.005	Maternal smoking; tobacco smoke exposure (0–4 years and at 10 years); SES; gender; BMI; current smoking status; exposure to smoke outside the home	**	14.16	**↓**
		1–9/hour	–0.02	0.04	0.6			*0.0002*	
		10/day	0.01	0.05	0.7				
		Seldom	0.02	0.03	0.5				
cg03476673 (*n* = 329)	CRISPLD2	>10/hour	–0.23	0.07	0.0009	Maternal smoking; tobacco smoke exposure (0–4 years and at 10 years); SES; gender; BMI; current smoking status; exposure to smoke outside the home	*** ^	9.07	**↓**
		1–9/hour	–0.12	0.07	0.09			*0.003*	
		10/day	–0.11	0.08	0.2				
		Seldom	–0.08	0.06	0.2				
cg07023532 (*n* = 362)	ACOT4	>10/hour	0.18	0.05	0.0006	Gender; BMI	***	4.39	**↑**
		1–9/hour	0.23	0.05	<.0001		***	*0.04*	
		10/day	0.21	0.06	0.0008		***		
		Seldom	0.14	0.05	0.003		**		
cg20255272 (*n* = 348)	VWA1	>10/hour	0.24	0.07	0.0009	Tobacco smoke exposure (0–4 years only); gender; BMI; SES; current smoking status	**	13.83	**↑**
		1–9/hour	0.13	0.07	0.08			*0.0002*	
		10/day	0.08	0.09	0.3				
		Seldom	0.001	0.06	1.0				
cg12417992 (*n* = 362)	SLC6A9	>10/hour	0.12	0.04	0.001	Maternal smoking; tobacco smoke exposure (0–4 years only); SES; gender	**	7.91	**↑**
		1–9/hour	0.11	0.04	0.003		* ^	*0.005*	
		10/day	0.10	0.04	0.02				
		Seldom	0.04	0.03	0.2				
cg04154465 (*n* = 351)	WNT2B	>10/hour	0.23	0.06	0.0003	SES; gender; BMI	***	16.12	**↑**
		1–9/hour	0.17	0.06	0.008		**	*<0.0001*	
		10/day	0.11	0.08	0.2				
		Seldom	0.02	0.06	0.7				
cg12813768 (*n* = 336)	SYCP1	>10/hour	–0.34	0.09	0.0001	Maternal smoking; tobacco smoke exposure (0–4 years and at 10 years); SES; gender; current smoking status; exposure to smoke outside the home	***	16.56	**↓**
		1–9/hour	–0.18	0.09	0.04		*	*<0.0001*	
		10/day	–0.08	0.11	0.5				
		Seldom	–0.08	0.08	0.3				
cg14162906 (*n* = 362)	TMEM222	>10/hour	0.11	0.04	0.004	BMI; gender	**	5.45	**↑**
		1–9/hour	0.13	0.04	0.002		**	*0.02*	
		10/day	0.12	0.05	0.01		*		
		Seldom	0.04	0.04	0.2				
cg24361098 (*n* = 362)	BCL11A	>10/hour	0.25	0.06	<.0001	BMI; gender	***	10.56	**↑**
		1–9/hour	0.15	0.06	0.02		*	*0.001*	
		10/day	0.15	0.07	0.04		*		
		Seldom	0.13	0.05	0.02		*		
cg16147794 (*n* = 329)	SLC16A10	>10/hour	–0.19	0.07	0.006	Maternal smoking; tobacco smoke exposure (0–4 years and at 10 years); SES; gender; BMI; current smoking status; exposure to smoke outside the home	**	8.91	**↓**
		1–9/hour	–0.07	0.07	0.3			*0.003*	
		10/day	–0.13	0.08	0.1				
		Seldom	0.06	0.06	0.4				
cg16668397 (*n* = 362)	JPH3	>10/hour	0.13	0.04	0.002	BMI; gender	**	10.56	**↑**
		1–9/hour	0.13	0.04	0.003		**	*0.001*	
		10/day	0.07	0.05	0.2				
		Seldom	0.03	0.04	0.4				
cg26419883 (*n* = 362)	TRPM5	>10/hour	0.15	0.04	0.0003	BMI; gender	***	13.64	**↑**
		1–9/hour	0.06	0.04	0.2			*0.0003*	
		10/day	0.05	0.05	0.3				
		Seldom	0.03	0.04	0.5				
cg21775675 (*n* = 329)	TMEM161B	>10/hour	–0.17	0.05	0.001	Maternal Smoking; Tobacco Smoke Exposure (0–4 years and at 10 years); SES; gender; BMI; current smoking status; exposure to smoke outside the home	**	10.07	**↓**
		1–9/hour	–0.05	0.05	0.3			*0.002*	
		10/day	–0.09	0.06	0.2				
		Seldom	0.002	0.05	1.0				
cg04794690 (*n* = 362)	PADI3	>10/hour	0.18	0.05	0.0005	BMI; gender	***	11.27	**↑**
		1–9/hour	0.22	0.05	<.0001		***	*0.0009*	
		10/day	0.08	0.06	0.2				
		Seldom	0.12	0.05	0.008		**		
cg06942649 (*n* = 362)	FBXO25	>10/hour	0.26	0.08	0.002	BMI; gender	**	7.5	**↑**
		1–9/hour	0.26	0.09	0.002		**	*0.007*	
		10/day	0.22	0.10	0.03		*		
		Seldom	0.09	0.08	0.2				
cg18459806 (*n* = 329)	NIN	>10/hour	–0.16	0.04	<.0001	Maternal smoking; tobacco smoke exposure (0–4 years and at 10 years); SES; gender; BMI; current smoking status; exposure to smoke outside the home	***	13.46	**↓**
		1–9/hour	–0.07	0.04	0.07		*	*0.0003*	
		10/day	–0.10	0.05	0.04		*		
		Seldom	–0.03	0.04	0.4				
cg20631351 (*n* = 362)	PALM	>10/hour	0.12	0.04	0.001	BMI; gender	**	10.25	**↑**
		1–9/hour	0.10	0.04	0.01		*	*0.002*	
		10/day	0.07	0.05	0.2		^		
		Seldom	0.03	0.04	0.3		^		
cg00347824 (*n* = 362)	NSMAF	>10/hour	0.19	0.05	0.0003	BMI; gender	***	8.63	**↑**
		1–9/hour	0.09	0.05	0.09			*0.004*	
		10/day	0.12	0.06	0.05				
		Seldom	0.06	0.05	0.2				
cg17053854 (*n* = 348)	SEPT9	>10/hour	0.09	0.03	0.003	Tobacco smoke exposure (at 10 years); BMI; gender	**	13.15	**↑**
		1–9/hour	0.09	0.03	0.003		**	*0.0003*	
		10/day	0.04	0.04	0.3				
		Seldom	0.01	0.03	0.8				
cg25324786 (*n* = 336)	RASA3	>10/hour	0.15	0.05	0.005	Maternal smoking; tobacco smoke exposure (0–4 years and at 10 years); SES; gender; current smoking status; exposure to smoke outside the home	** ^	17.15	**↑**
		1–9/hour	0.09	0.05	0.09			*<.0001*	
		10/day	–0.03	0.06	0.7				
		Seldom	–0.03	0.05	0.5				
cg26720961 (*n* = 336)	TSNARE1	>10/hour	0.12	0.06	0.05	Maternal smoking; tobacco smoke exposure (0–4 years and at 10 years); SES; gender; current smoking status; exposure to smoke outside the home	^	9.55	**↑**
		1–9/hour	0.03	0.06	0.7			*0.002*	
		10/day	–0.01	0.08	0.9				
		Seldom	–0.10	0.06	0.09				
cg05575058 (*n* = 329)	FAM164A	>10/hour	–0.16	0.05	0.001	Maternal smoking; tobacco smoke exposure (0–4 years and at 10 years); SES; gender; BMI; current smoking status; exposure to smoke outside the home	**	7.77	**↓**
		1–9/hour	–0.08	0.05	0.09			*0.006*	
		10/day	–0.10	0.06	0.1				
		Seldom	–0.06	0.04	0.2				
cg15742605 (*n* = 329)	SAMD11	>10/hour	0.19	0.05	0.0003	Maternal smoking; tobacco smoke exposure (0–4 years and at 10 years); SES; gender; BMI; current smoking status; exposure to smoke outside the home	***	18.74	**↑**
		1–9/hour	0.08	0.05	0.1			*<.0001*	
		10/day	0.04	0.06	0.5				
		Seldom	–0.01	0.05	0.9				
cg26185508 (*n* = 362)	CDCP2	>10/hour	0.15	0.05	0.002	BMI; gender	**	5.48	**↑**
		1–9/hour	0.18	0.05	0.0005		***	*0.02*	
		10/day	0.17	0.06	0.005		**		
		Seldom	0.07	0.04	0.1				
cg02378006 (*n* = 329)	UNC5B	>10/hour	0.19	0.06	0.001	Maternal smoking; tobacco smoke exposure (0–4 years and at 10 years); SES; gender; BMI; current smoking status; exposure to smoke outside the home	**	15.56	**↑**
		1–9/hour	0.07	0.06	0.2			*<.0001*	
		10/day	0.001	0.07	1.0				
		Seldom	–0.0001	0.05	1.0				
cg08462127 (*n* = 329)	MYOM2	>10/hour	0.13	0.05	0.006	Maternal smoking; tobacco smoke exposure (0–4 years and at 10 years); SES; gender; BMI; current smoking status; exposure to smoke outside the home	** ^	6.48	**↑**
		1–9/hour	0.04	0.05	0.4			*0.01*	
		10/day	0.11	0.06	0.06				
		Seldom	–0.02	0.04	0.7				
cg11017318 (*n* = 329)	SYT16	>10/hour	–0.06	0.04	0.1	Maternal smoking; tobacco smoke exposure (0–4 years and at 10 years); SES; gender; BMI; current smoking status; exposure to smoke outside the home		1.37	NA
		1–9/hour	–0.05	0.04	0.2			*0.2*	
		10/day	–0.08	0.05	0.1				
		Seldom	0.0001	0.04	1.0				

Once the Dunnett’ tests provided statistical evidence of differences in marginal means of the heavy vehicular traffic frequency, a second test for trend is performed to assess a ‘dose–response’ relationship.

*** *P* < 0.001;

** *P* < 0.01;

* *P* < 0.05; *P* < 0.1.

**Figure 2:**
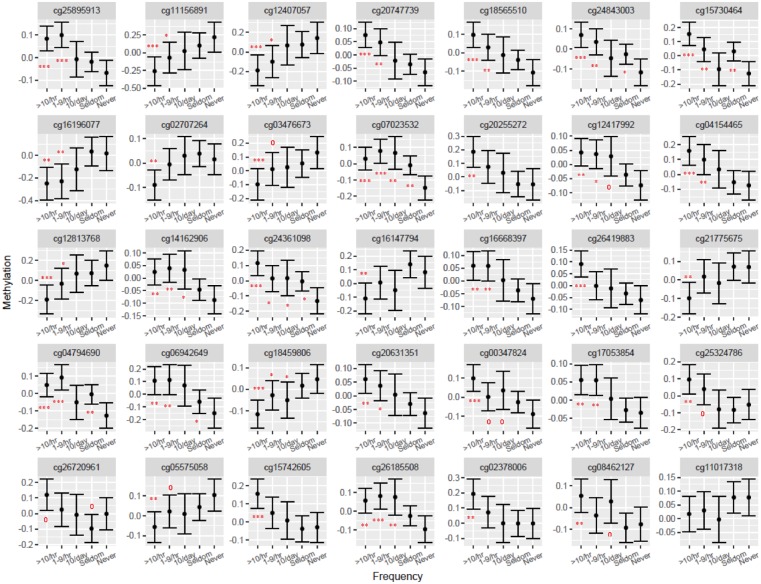
Adjusted means (with 95% confidence limits) for DNA-M of 35 significant CpG sites associated with the frequency of heavy vehicles that passed by subjects’ homes for a subset of the IoW birth cohort, F1 generation (range of sample size: 336–369). Adjustments depended on CpG site under consideration and included: Maternal Smoking; Tobacco Smoke Exposure (0–4 years and/or at 10 years); SES; BMI; gender; current smoking status; exposure to smoke outside the home. ***P < 0.001; **P < 0.01; *P < 0.05; *P* < 0.1. Once the Dunnett’ tests provided statistical evidence of differences in marginal means of the heavy vehicular traffic frequency, a second test for trend is performed to assess a ‘dose–response’ relationship

In particular, we found 23 CpGs that were more differentially methylated (Fig. 2). Nineteen of these 23 CpG sites are found in the body of the associated genes while the remaining 4 are located in promoter regions (TSS1500 and TSS200) (Table 3). Conversely of the 11 CpGs that were less methylated with increasing heavy vehicle traffic frequency (Fig. 2), 5 are located in the body of the gene, an additional 5 are found in promoter regions and the last one is ∼50 kb upstream of *TMEM161B* (Table 3). Among subjects reporting the two highest heavy vehicle traffic frequencies: 1–9 per hour or >10 per hour, statistical significance was consistently reached for the differential methylation observed at these CpG sites (*P* ≤ 0.05, Table 6).

Stratification by current smoking status, revealed similar trends among smokers and nonsmokers. Although statistically significant differences were only detected for 12 CpGs among smokers and 26 CpGs among nonsmokers, mainly for those reporting >10 heavy vehicles per hour (Supplementary Tables S2 and S3, respectively). Regression results for males only revealed only 10 statistically significant CpG sites with differential methylation: 7 were more methylated and 3 were less methylated (Supplementary Table S4). Results for females indicated 31 significant CpGs with 22 more methylated and 9 less methylated (Supplementary Table S5). The direction of methylation remained the same and the smaller number of significant CpG sites among male subjects is probably due to their smaller sample size in this birth cohort (*n* = 124).

### Results of Replication and Gene Expression Analysis

We replicated the findings for 31 of 35 CpG sites identified in a smaller sample of 140 newborns in the F2 generation. Two CpG sites: cg25895913 (*LGI2*) and cg00347824 (*NSMAF*) were associated with traffic frequencies, and the direction of the effect was the same as in the F1 subset. The former CpG site had less methylation, while the latter had more methylation, with increasing vehicular traffic frequency respectively (Table 7). Then, Spearman rank correlation analysis revealed seven CpG sites: cg24843003 (*DAZAP1*), cg03476673 (*CRISPLD2*), cg12417992 (*SLC6A9*), cg04154465 (*WNT2B*), cg24361098 (*BCL11A*), cg16668397 (*JPH3*) and cg17053854 (*SEPT9*) whose differential methylation was significantly correlated with gene expression (Table 8, partial *r* ≤ 0.27, *P*-value ≤ 0.05). For an additional two of these CpG sites: cg14162906 (*TMEM222*) and cg17053854 (*SEPT9*), there were marginal correlations with expression data from their associated genes (Table 8, 0.05 > *P*-value ≤ 0.06).

**Table 7:** Results for linear models for CpG sites associated with the frequency of vehicles passing by homes of study subject during pregnancy

CpG	Associated gene	Heavy vehicle frequency (ref = never)	Estimate	Standard error	*P* value	Significant covariates in final model	Dunnett's test (LSMEAN = never)	Linear trend test (*F* value, df = 1) *P value*	Direction of methylation in F2 generation	Direction of methylation in F1 generation
cg15730464 (*n* = 140)	LGI2	>10/hour	–0.02	0.01	0.072	Gender; birthweight		3.22	**↓**	**↓**
		1–9/hour	–0.02	0.01	0.040		0	*0.07*		
		10/day	–0.02	0.01	0.095					
		Seldom	–0.02	0.01	0.118					
cg00347824 (*n* = 140)	NSMAF	>10/hour	0.02	0.01	0.018	Gender; birthweight	*	7.36	**↑**	**↑**
		1–9/hour	0.02	0.01	0.017		*	*0.008*		
		10/day	0.01	0.01	0.032		0			
		Seldom	0.01	0.01	0.120					

Once the Dunnett’ tests provided statistical evidence of differences in marginal means of the heavy vehicular traffic frequency, a second test for trend is performed to assess a ‘dose–response’ relationship.

*** *P* < 0.001;

** *P* < 0.01;

* *P* < 0.05; *P* < 0.1.

**Table 8:** Partial Spearman rank correlation coefficients between DNA-m and gene expression in the cord blood of 155 newborns born to a subset of the 369 subjects from the IoW cohort

CpG	Direction of methylation	Associated gene	Gene expression	Partial correlation coefficient (Spearman)	*P* value
cg25895913	** ↑**	CDH4	A_21_P0009987	–0.09	0.29
cg25895913	** ↑**	CDH4	A_23_P17593	0.01	0.92
cg25895913	** ↑**	CDH4	A_33_P3310976	–0.07	0.40
cg20747739	** ↑**	FAM132A	A_32_P75792	–0.01	0.91
cg18565510	** ↑**	ACAP3	A_33_P3230290	0.01	0.93
cg18565510	** ↑**	ACAP3	A_33_P3398564	0.04	0.61
cg24843003	** ↑**	DAZAP1	A_23_P165247	0.19	**0.021**
cg24843003	** ↑**	DAZAP1	A_33_P3359590	–0.18	**0.028**
cg15730464	**↓**	LGI2	A_33_P3358397	–0.04	0.59
cg15730464	**↓**	LGI2	A_33_P3393655	0.03	0.70
cg16196077	**↓**	RTKN2	A_21_P0007001	0.02	0.78
cg16196077	**↓**	RTKN2	A_21_P0007002	–0.09	0.30
cg16196077	**↓**	RTKN2	A_24_P13041	0.06	0.45
cg16196077	**↓**	RTKN2	A_32_P471485	–0.04	0.61
cg16196077	**↓**	RTKN2	A_33_P3219527	–0.09	0.25
cg02707264	**↓**	MYRIP	A_23_P326760	0.05	0.58
cg03476673	**↓**	CRISPLD2	A_23_P106602	–0.20	**0.015**
cg12417992	** ↑**	SLC6A9	A_21_P0001726	–0.02	0.85
cg12417992	** ↑**	SLC6A9	A_21_P0001727	–0.04	0.61
cg12417992	** ↑**	SLC6A9	A_23_P11984	0.27	**0.0011**
cg12417992	** ↑**	SLC6A9	A_33_P3402615	0.06	0.45
cg04154465	** ↑**	WNT2B	A_23_P138352	0.16	**0.0495**
cg12813768	**↓**	SYCP1	A_23_P722	–0.02	0.83
cg14162906	** ↑**	TMEM222	A_23_P97442	0.09	0.30
cg14162906	** ↑**	TMEM222	A_33_P3259722	0.15	***0.0622***
cg24361098	** ↑**	BCL11A	A_21_P0002443	0.08	0.33
cg24361098	** ↑**	BCL11A	A_21_P0002444	–0.10	0.24
cg24361098	** ↑**	BCL11A	A_21_P0002445	–0.10	0.22
cg24361098	** ↑**	BCL11A	A_24_P402588	0.11	0.17
cg24361098	** ↑**	BCL11A	A_24_P411186	0.26	**0.0012**
cg24361098	** ↑**	BCL11A	A_33_P3249589	–0.01	0.91
cg24361098	** ↑**	BCL11A	A_33_P3249595	–0.06	0.44
cg16147794	**↓**	SLC16A10	A_24_P98047	0.08	0.31
cg16147794	**↓**	SLC16A10	A_33_P3308512	0.07	0.43
cg16668397	** ↑**	JPH3	A_21_P0008991	0.07	0.37
cg16668397	** ↑**	JPH3	A_21_P0008992	0.16	*0.05*
cg16668397	** ↑**	JPH3	A_21_P0008993	0.20	**0.015**
cg16668397	** ↑**	JPH3	A_21_P0008994	–0.01	0.88
cg16668397	** ↑**	JPH3	A_24_P150791	0.16	**0.048**
cg16668397	** ↑**	JPH3	A_33_P3423721	–0.20	**0.016**
cg26419883		TRPM5	A_23_P87279	–0.03	0.71
cg21775675	**↓**	TMEM161B	A_21_P0000876	–0.02	0.83
cg21775675	**↓**	TMEM161B	A_21_P0004517	–0.05	0.54
cg21775675	**↓**	TMEM161B	A_23_P156355	–0.04	0.60
cg04794690	** ↑**	PADI3	A_23_P126869	0.00	0.97
cg06942649	** ↑**	FBXO25	A_21_P0005641	–0.06	0.49
cg06942649	** ↑**	FBXO25	A_21_P0005919	–0.01	0.92
cg06942649	** ↑**	FBXO25	A_21_P0010595	0.07	0.40
cg06942649	** ↑**	FBXO25	A_21_P0012049	0.09	0.30
cg06942649	** ↑**	FBXO25	A_21_P0013463	0.09	0.30
cg06942649	** ↑**	FBXO25	A_21_P0013464	–0.01	0.93
cg06942649	** ↑**	FBXO25	A_21_P0013530	0.07	0.42
cg06942649	** ↑**	FBXO25	A_21_P0013531	0.01	0.92
cg06942649	** ↑**	FBXO25	A_23_P94159	0.04	0.65
cg06942649	** ↑**	FBXO25	A_33_P3841368	0.04	0.60
cg18459806	**↓**	NIN	A_23_P396353	0.06	0.47
cg18459806	**↓**	NIN	A_24_P412512	0.02	0.84
cg00347824	** ↑**	NSMAF	A_23_P134809	0.01	0.88
cg17053854	** ↑**	SEPT9-	A_21_P0009276	–0.11	0.17
cg17053854	** ↑**	SEPT9-	A_21_P0009277	–0.15	***0.06***
cg17053854	** ↑**	SEPT9-	A_21_P0009278	–0.07	0.37
cg17053854	** ↑**	SEPT9-	A_21_P0009279	–0.20	**0.014**
cg17053854	** ↑**	SEPT9-	A_21_P0009280	–0.01	0.92
cg17053854	** ↑**	SEPT9-	A_21_P0009396	–0.13	0.13
cg25324786	** ↑**	RASA3	A_21_P0008023	–0.01	0.89
cg25324786	** ↑**	RASA3	A_33_P3262515	–0.05	0.56
cg26720961	** ↑**	TSNARE1	A_21_P0005916	0.00	0.96
cg26720961	** ↑**	TSNARE1	A_33_P3291567	–0.06	0.44
cg26720961	** ↑**	TSNARE1	A_33_P3297126	–0.09	0.30
cg15742605	** ↑**	SAMD11	A_21_P0001250	–0.06	0.45
cg15742605	** ↑**	SAMD11	A_33_P3818959	–0.02	0.77
cg26185508	** ↑**	CDCP2	A_33_P3259522	–0.05	0.51
cg02378006	** ↑**	UNC5B	A_23_P52336	0.04	0.61
cg02378006	** ↑**	UNC5B	A_32_P52153	0.13	0.11
cg08462127	** ↑**	MYOM2	A_21_P0005646	–0.07	0.39
cg08462127	** ↑**	MYOM2	A_23_P258912	0.04	0.64
cg11017318	**↓**	SYT16	A_24_P143324	0.02	0.85

*P*-values in bold and bold italics denote statistical significance less than or equal to 0.05 and 0.1 respectively.

## Discussion

We aimed to answer two questions: (i) *Which specific CpG sites are associated with heavy vehicular traffic in the birth cohort?* (ii) *Are there any trends in the association between differential DNA-m and the frequency of heavy vehicular traffic?* Regarding the first question, we found 35 CpG sites to be associated with heavy vehicular traffic. These CpG sites were associated with 34 different genes (two CpG sites—cg11156891 and cg12407057 mapped to the same gene: *ANKRD65*). Additionally, 31 of these genes have been reported to be associated with air pollution related chemicals such as benzo(a)pyrene in the comparative toxicogenomics database. In adopting an epigenome-wide approach, as opposed to a candidate gene approach, our analysis adds novel information on epigenetic markers for traffic-related air pollution exposure. These exposure-associated changes in the epigenome could be used to identify exposure to air pollutants, particularly those from incomplete combustion of fuels such as diesel which is often used in buses and trucks. With further research, it can also guide the development of effective clinical and public health interventions and reduce the burden of air pollution–related health outcomes.

For the second question on assessing the association between differential DNA-m and traffic-related air pollution, we found 23 CpGs that were more methylated, and 11 CpGs that were less methylated with increasing heavy vehicular traffic frequency for all subjects after adjusting for confounders. These associations between heavy vehicular traffic frequency and DNA-m measurements persisted after stratification by current smoking status for 26 and 12 CpG sites among nonsmokers and smokers, respectively. Among subjects reporting the two highest heavy vehicular frequency levels: 1–9 per hour or >10 per hour, statistical significance was consistently reached for the differential methylation observed at these CpG sites (*P* ≤ 0.05, Table 6). This exploratory study highlights the fact that epigenetic differences can be observed among subjects exposed to varying frequencies of local traffic.

Our results suggest that exposure to emissions, presumably from the exhaust of heavy vehicles passing by the residences of study subjects, may have an impact on DNA-m. It has been suggested that epigenetic states can convey susceptibility to air pollution, which can lead to biological changes, and ultimately, adverse health [43, 52]. DNA-m profiles can provide insight into aspects of biology such as gene activity and regulation, and our gene enrichment analysis offers examples of how the genes associated with the CpG sites are related to various molecular functions, pathways and some rare diseases. Based on the location of the CpG site such as promoter or body, altered methylation may lead to increased transcription, silencing or altered splicing [53–56]. Hence, a differential transcription level is only one of the consequences of DNA-m. For instance, it has been considered that methylation in promoter regions may lead to changes in gene expression, i.e. gene silencing [57]; and such changes can serve as putative markers or risk factors for altered susceptibility and/or disease states. Additionally, DNA-m can help in identifying CpG sites, and possibly genes, that are more susceptible to environmental exposures [58].

In a replication and gene expression analysis study among 140 newborns from the F2 generation, 6 of the 7 CpG sites that correlated with expression, cg24843003 (*DAZAP1*), cg12417992 (*SLC6A9*), cg04154465 (*WNT2B*), cg24361098 (*BCL11A*), cg16668397 (*JPH3*) and cg17053854 (*SEPT9*), are located in the bodies of the associated genes. The seventh, cg03476673, is found in the 5′UTR region of *CRISPLD2.* Of the remaining 23 CpG sites with corresponding expression data but no statistically significant correlations, 3 are located in the TSS1500 region including cg14162906 (*TMEM222*) which achieved a marginal significance. The rest are in the following regions: body of the associated gene (*n* = 14), TSS200 region (*n* = 2), 5′UTR region (*n* = 2), ∼50 kb upstream of *TMEM161B* (*n* = 1) and ∼200 kb upstream of *SYT16* (*n* = 1). The association of 31 of 34 genes (identified from CpG sites in this study) to air pollution–related chemicals adds plausibility to potential environment–gene interactions, and can contribute to emerging data that provide a more complete view of environmental exposures. We posit that traffic-related air pollution may be a plausible environmental exposure of interest on the IoW.

With increasing evidence that exposure to air pollution is associated with adverse health outcomes, biologically plausible mechanistic pathways of air pollution’s effects, such as oxidative stress, inflammation, coagulation, endothelial function and hemodynamic response, have been implicated [59]. Exposure to ambient particulate matter, which is known to be emitted from diesel truck traffic, is associated with decreased lung function and increases in respiratory disease and symptoms such as asthma exacerbation [47, 60–62, 72]. Exposure to gaseous air pollutants including nitrogen species (e.g. NO_2_, NO, NOx) are also associated with deleterious effects such as bronchial reactivity, airway oxidative stress, pulmonary and systemic inflammation [63–66]. Several epidemiologic studies have reported that short-term increases in ambient pollutants such as PM_2.5_ and nitrogen dioxide (NO_2_) are associated with increases in airway inflammation in children and adults [67–73].

A recent epigenome-wide meta-analysis by Gruzieva et al. [74] provides evidence on the association between prenatal air pollution exposures and differences in the methylation of several genes in cord blood. In particular, the authors found significant associations between NO_2_ exposures and DNA-m for CpG sites that mapped to genes in the solute carrier family (*SLC*), family with sequence similarity *(FAM*) and transmembrane proteins (*TMEM*). Five CpG sites [associated gene in square brackets] (cg12417992 [*SLC6A9*], cg14162906 [*TMEM222*], cg16147794 [*SLC16A10*], cg20747739 [*FAM132A*], cg21775675 [*TMEM161B*]) related to three gene superfamilies from the Gruzieva et al. meta-analysis were associated with heavy vehicular traffic frequency and DNA-m in our study. The association of one of the CpG sites with *FAM132A* (codes for an important anti-inflammatory adipokine [75]), strengthens the hypothesis that inflammation may be a possible mechanism though which ambient air pollution affects human health [76].

While underlying molecular alterations of air pollution mediated adverse health remain to be further investigated, another recent study with two European cohorts identified decreasing DNA-m on CpG island shores, shelves and gene bodies with increasing concentrations of nitrogen oxide (NO) species [77]. NO species are currently the best available indicators of spatial variation and mixtures of outdoor urban air pollution such as traffic [78]. Our analysis did not reveal CpG sites associated with the inflammatory genes mentioned in the above study, and to the best of our knowledge, the significant CpG sites reported in our study have not been reported in previous air pollution studies. This may be due to differences in (i) study populations, (ii) exposure assessment and concentrations, (iii) complex multiple biological pathways or (iv) a combination of any of the previous three reasons. These newly identified CpG sites and associated genes are certainly worth exploring in larger cohorts.

In our study, the two CpG sites that were associated with vehicular traffic in both the F1 and F2 generation may be reflective of the effects of TRAP exposures at these two loci. It also suggests possible prenatal exposures to traffic-related air pollutants in the F2 generation. Secondly, correlation between DNA-m and gene expression at 7 of 31 CpG sites (and three marginal correlations) supports the hypothesis that DNA-m is a potential mechanism through which traffic-related air pollutants can affect gene expression. Three of these seven CpG sites are associated with genes previously identified in the literature to be related to inhalation. For instance, *CRISPLD2* has been identified as a glucocorticoid responsive gene that modulates cytokine function in airway smooth muscle cells [79]. *WNT2B* has been reported to be associated with embryonic origins of the lung since the inactivation of *WNT2A* and *WNT2B*, resulted in complete absence of lung development [80]. Methylation of *JPH3* from sputum samples is a sensitive and specific predictor of chronic mucous hypersecretion in former male smokers [81]. The lack of 100% replication and correlation in our analysis may be due to small sample sizes and exposure misclassification from the use of questionnaire data rather than air pollution data (for instance the questionnaire administered at 18 years specified ‘heavy vehicle’ while the questionnaire during pregnancy only mentioned ‘vehicle’). While our results must be interpreted with caution, there are additional studies that add to the evidence that adverse effects of air pollution that can occur when one is exposed. A recent study, which did not replicate its results in a separate independent cohort, found that living close to major roadways at birth was associated with differential cord blood methylation [82]. Another study, which was also not replicated in an independent cohort found signiﬁcant associations between long-term air pollution exposure (NO_2_) and DNA-m for seven CpG sites (Bonferroni corrected threshold *P* < 1.2E–7) [83].

With continuing indication that exposure to ambient air pollutants may contribute to adverse public health [1, 84], further research is needed to identify the components of air pollution that determine its toxicity and a pristine environment such as the IoW could offer a suitable environment to study ambient air pollutant toxicity. The constituents of the pollution potentially generated by heavy vehicles may need to be identified so that early preventative and possible control strategies can be targeted efficiently. Whether these findings raise the risk for future cellular malfunction and disease is unknown. One main reason for the persistence (or the lack thereof) of such findings could be attributed to small sample sizes. In our case, the nonsmokers were consistently between 248 and 270 while smokers were between 78 and 95 subjects. Another reason could be due to the small magnitude effect sizes that are common with environmental epigenetic research [85]. Profiling of the epigenome over time in this population will help improve understanding of TRAP exposures and how the epigenome responds to this stimuli. Additionally, we found that secondhand smoke exposure is represented by the questions posed to subjects about tobacco smoke exposures since these variables were associated with the methylation of cg07555921 (*AHRR*), while the exposure variable was not. Therefore, these observed effects of heavy vehicular traffic on DNA-m may be without the contribution of this type of air pollution. Further studies in the future may be needed to examine this in depth.

There are some limitations to this study. First in this study, our exposure variable of interest, heavy vehicular traffic frequency, was ascertained by questionnaire responses from study subjects and we did not attempt to conduct exposure assessment inside or outside their residences, and these analyses were based on current residences (at the time of the blood draw at 18 years old in the F generation) as opposed to conditions in former places of residence. Secondly, the associations observed in this study are informative. However, further analysis may be needed to assess other self-reported exposures such as tobacco exposures, particularly on a cumulative scale. Given that the data in this pilot study are from a birth cohort to which a third generation follow-up has been added, further investigation of the DNA-m of the same subset of this population at earlier time points or in their offspring could address some of these limitations. Thirdly, methylation data were obtained from whole blood but not from specific cell subgroups, due to cost, but while differential methylation may or may not be present in all cell subsets, we believe that important biological insights still may be gained from studying DNA-m in whole blood [86]. Moreover, we did adjust for the cell types in the screening step of the analysis, thereby overcoming this limitation. Additionally, multiple studies have validated the 450K DNA-m array from Illumina [87–89], and this assay is generally accepted in the scientific literature. Hence we did not see a necessity to additionally test the results of specific CpGs from the 450K DNA-m array with methyl-specific qPCR. The use of bisulfite sequencing can be challenging, since it reduces genome complexity and some of the methods may not differentiate between methylcytosine and hydroxymethylcytosine. The incorporation of appropriate controls for bisulfite reactions and careful interpretation of DNA-m level after accounting for cell types can overcome some of these challenges [90]. An overview of major difficulties related to bisulfite sequencing and how to overcome them are presented in the review by Li et al. [91]. Although the correlations between CpG sites and expression data reached statistical significance, the coefficients were weak. One may consider this as a limitation of our study; however, gene expression is influenced by multiple factors and our analysis only focus on the role of DNA-m on gene expression. Future studies with large sample sizes need to further investigate associations between traffic-related DNA-m and gene expression, taking other factors such as genetic polymorphisms and network of related genes, into consideration. Finally, since this is the first study that shows an effect of varying heavy vehicular traffic frequency on DNA-m among residents on the Isle, further replication of these associations in an independent cohort is needed.

## Conclusions

Our findings reveal differences in DNA-m in participants who reported higher heavy vehicular traffic frequencies when compared with participants who reported lower frequencies. Such findings may be attributed to TRAP exposure and suggest that further studies are needed.

## Materials and Methods

### Study Population

Subjects in this study are from a whole population birth cohort established in 1989 on the IoW, UK, to prospectively study the natural history of allergies and asthma. This cohort has been previously described in detail elsewhere [92]. Informed consents and detailed information from questionnaires were obtained from participants at recruitment and at each follow-up year: 1, 2, 4, 10 and 18 years [93]. The questionnaires for the entire birth cohort study are for study-specific objectives, while asthma and allergy symptom questions are from the validated International Study of Asthma and Allergies in Childhood (ISAAC) [92]. Local Research Ethics Committees approved of the parent study, and the Institutional Review Board at the Medical University of South Carolina approved the current study. In this exploratory analysis, we focus on 369 individuals (245 women and 124 men) with DNA-m measurements at age 18 years. Due to the original study question of inheritance via females, we included more females than males at 18 years.

### DNA-m Analysis

DNA was extracted from peripheral blood samples and its concentration was determined by Qubit quantitation, as described previously [94]. Genome-wide DNA-m was assessed using the Illumina Infinium Human Methylation 450 beadchip (Illumina, Inc., CA, USA), which interrogates >484 000 CpG sites associated with approximately 24 000 genes. Arrays were processed and imaged using the manufacturer’s recommendations, as described elsewhere [95]. Multiple identical control samples were assigned to each bisulfite conversion batch, and the samples were randomly distributed on microarrays to assess assay variability and to control batch effects respectively.

Methylation levels (*β* values) were calculated for queried CpG loci using the methylation module of GenomeStudio software [96]. DNA-m levels for each CpG were estimated as the proportion of intensity of methylated (*M*) over the sum of methylated (*M*) and unmethylated (*U*) probes, *β* = *M*/[*c* + *M* + *U*] with *c* being a constant to prevent dividing by zero [97]. DNA-m levels were corrected for batch effect using ‘*IMA*’ and ‘*ComBat*’ packages in R [98]. *M*-values were calculated as log 2 ratio of the intensities of methylated probe versus unmethylated probe, and used in subsequent analysis [99]. The detection *P*-value for each CpG site was used as a quality control measure of probe performance and CpG sites with: (i) detection *P*-value > 0.01 in >10% of the samples and (ii) probe single nucleotide polymorphism (SNP) excluded from all analyses.

We estimated the proportion of cell types in adult peripheral blood using the estimateCellCounts() function in *minfi* package following the Houseman approach [100] using the adult reference panel [101].

### Exposure Assessment

The exposure variable of interest, the frequency of heavy vehicular traffic, was determined through questionnaire responses from the subjects to the question: ***How often do heavy vehicles (e.g. trucks/buses) pass your house or on the street less than 100 meters away?*** The five-point response included: never, seldom, 10 per day, 1–9 per hour or >10 per hour. We also assessed answers to other air pollution related questions such as ‘***How often do cars pass your house or on the street less than 100 meters away?’*** and ‘***How frequently are you annoyed by outdoor air pollution (from traffic industry, etc) in your home if you keep the window open?***’. All subjects were approximately 18 years old when the questionnaire containing these questions was administered. While we have not seen of any study in the literature that has used the same question to assess exposure to TRAP, others have used questionnaire-derived assessments as air pollution exposure variables [102, 103]. Others have used such questions along with proximity to roadways, air pollution measurements, land use regressions together with the validated and widely used International Study of Asthma and Allergies in Childhood (ISAAC) questionnaire to successfully characterize health effects of interest [104–106].

### Covariates of Interest

For this exploratory study, the covariates of interest obtained from the subjects’ mothers were as follows: (i) gender; (ii) maternal smoking status during pregnancy obtained from questionnaires at birth of the subject; (iii) tobacco smoke exposure obtained through questionnaires completed at birth and at ages 1, 2, 4 and 10 years. Other covariates were obtained from the questionnaire administered to the subjects at age 18: (iv) socio-economic status (SES) ascertained from the question ‘what is your family’s annual income (estimate)?’; (v) current smoking status, and age subject started to smoke if applicable; (vi) exposure to smoke outside the home ascertained by the question ‘are you regularly exposed to smoking outside the home?’; (vii) BMI calculated from height and weight measurements obtained during the 18-year follow-up, using the following formula: weight (kg)/height (m)^2. In addition, we considered the type of residential property the subjects lived in (rented privately, rented council/housing association, owned privately or other), whether the subjects were still living with their parents, and the duration of living in the present house (obtained in the course of the 4-year follow-up).

### Statistical Analysis

Descriptive statistics and chi square tests were used to assess whether the 369 subjects in this study were representative of the total birth cohort. Then, we conducted statistical analyses in two main steps:

#### Step 1: Epigenome-wide Association Analysis

**Screening tool.** We employed *ttScreening* package (an epigenome-wide DNA-m sites screening tool) to examine CpGs that are potentially associated with the frequency of heavy vehicles passing by subjects’ homes at age 18 years. This approach to screen epigenome-wide data was used since it generally performs better and has the potential to control both types I and II errors [107]. Specifically, the *ttScreening* package conducts surrogate variable analysis, unexplained variation in the data is removed, prior to an iterative training-testing procedure. This training-testing method performs better than methods such as the FDR and the Bonferroni in reducing false-positive and false-negative results. In addition to providing internal validation, the use of training-testing builds more generalized models than those constructed by traditional methods, and can detect additional loci undetectable using traditional methods [107].

The analytical methods implemented in the package employed a screening process that filtered non-informative CpGs through 100 iterations of a training-and-testing (TT) process with robust regressions. We followed the default settings for the *ttScreening* method: (i) 2 of 3 of the data for training, (ii) the ‘two-step’ method for surrogate variable analysis (*sva.method*) [108], (iii) 100 iterations for the total number of screenings (*iterations*), (iv) 50% as the cutoff proportion of those 100 iterations (*cv.cutoff*) and (v) 0.05 significance level for the training (*train.alpha*) and testing data (*test.alpha*). The 100 iterations are recommended by the authors of the *ttScreening* package to create a balance between computing efficiency and adequate resampling to arrive at true associations. Also 50% is the default for the cutoff proportion since the informative CpGs are usually sparse in comparison to the candidate CpG sites, and the authors’ simulations identified 50% cutoff percentage as suitable for small and large sample sizes [107].

The independent and dependent variables were heavy vehicular traffic frequency and DNA-m, respectively. A CpG was selected as an informative site if it showed statistical significance in at least 70% of iterations. The *ttScreening( )* function automatically adjusts for multiple testing using three methods, including FDR, Bonferroni and the TT method [109].

**CpG by CpG analysis.** As an alternative to the *ttscreening* method, we also conducted multiple linear regressions with the *M* values of each CpG while adjusting for all covariates selected apriori and calculated adjusted *P*-values for the multiple comparisons (*p.adjust ( )* command in base R). The exposure variable in this case was classified as ‘Any’ versus ‘No’ heavy vehicular traffic frequency. All procedures in Step 1 were conducted with R (version 3.4.2) [110].

**Tobacco smoke exposure.** Prior epigenome-wide association studies have shown that the methylation of cg05575921 located in the *AHRR* gene is a robust indicator of tobacco smoke exposure [111, 112]. Even in different demographics, smoking histories and rates of false-negative self-report of smoking behavior, this CpG site can reliably detect smoking status [113]. Additionally, a recent study revealed that high levels of recent secondhand smoke exposure was inversely associated with DNA-m of cg05575921 in monocytes from nonsmokers, although the effects were weaker when compared with active smokers [114]. Hence we conducted linear regression models with self-reported smoking status and secondhand smoke exposures to examine the relationships between this CpG site and tobacco smoke exposure, as well as our exposure variable: heavy vehicular traffic frequency.

#### Step 2: Associations between the Frequency of Heavy Vehicular Traffic and DNA-m

To investigate preliminary associations with heavy vehicular traffic frequency, we assessed differences in unadjusted DNA-m of the CpGs identified in the *ttscreening* method in Step 1 using analysis of variance (ANOVA) on only heavy vehicular frequency. Then, the CpGs were further tested in multiple linear models that included potential confounders to assess their association with the heavy vehicular traffic frequency. A general form of the model is seen in Equation (1):

(1) $$DNA.M_{iv}=\;\alpha +\beta_{v}{+\;\gamma Covariate}_{i}+\;\varepsilon_{iv}$$

$where\;DNA.M_{iv}$ refers to the DNA-m for the *i*th subject reporting *v*th category of heavy vehicular frequency, *α* is the intercept and *ε* is the error term. The coefficient $\beta_{v}$ is the deviation of grand mean for *v*th category of heavy vehicle traffic frequency (seldom, 10 per day, 1–9 per hour and >10 per hour) compared to *never*. The *lsmeans* statement was used to derive model adjusted means.

### Modeling and Variable Selection

For a covariate to be considered a confounder, the estimate of the regression coefficient for heavy vehicle traffic frequency in the reduced model (that excluded the confounder of interest) had to fall outside the range of 10% of the estimate of the full model (the full model includes all covariates considered apriori in this study) [115]. The final models for each CpG site included gender and any identified confounders. Models were assessed for all subjects and then stratified by gender and current smoking status since exposure to tobacco can lead to extensive genome-wide changes in DNA-m [116].

### Adjusted DNA-m Means and Trend Test

We performed Dunnett’s tests to compare model adjusted (marginal) means from four heavy vehicle traffic frequency categories (seldom, 10 per day, 1–9 per hour or >10 per hour) against a control group mean (never) to check for statistically significant differences. We also used PROC IML’s ORPOL function in SAS [117] to obtain appropriate coefficients for contrast statements to test for linear trends in increasing heavy vehicular frequency with increasing or decreasing DNA-m measurements, only when marginal means were significantly different from the control mean (never category). When marginal means did not significantly differ from the control category, the results were not provided. *P* values <0.1 were considered statistically significant for the trend tests. Finally, marginal means for DNA-m were plotted by category of reported heavy vehicle traffic frequency. Step 2 was performed with the SAS statistical package (version 9.4; SAS Institute, Cary, NC, USA). All plots were derived using ‘ggplot’ function in R.

## Replication and Gene Expression

### Study Population

Thirty-one of 35 significant CpG sites found in the present study for the 369 subjects in the F1 generation were tested in the DNA-m and gene expression data from cord blood in the newborn cohort, the F2 generation (*n* = 155, born 2006–2013). This step constitutes a replication of the CpGs in a semi-independent cohort. In the F2 generation, there were 76 males and 79 females and the average birthweight was 3459.3 g (standard deviation: 504.6). The median birthweight was 3515 g (*n* = 148). The exposure variable was obtained from the questionnaire administered to the mothers during pregnancy. The mothers’ answers to this question were used as the exposure (independent) variable of interest: How often do vehicles pass your house or on the street less than 100 meters away? The answers were never, seldom, 10 per day, 1–9 per hour or >10 per hour. When a mother answered the question once instead of three times, this answer was assigned as the frequency of vehicles that passed by the home during the entire pregnancy. If she answered two or three times, the lowest frequency was assumed to be her exposure. This was to be conservative on their exposures since this pregnancy questionnaire did not specify ‘heavy vehicles’, compared to the question posed to them (F1 generation) at age 18. It also allowed for a distribution of responses as follows: never (2), seldom (8), 10 per day (26), 1–9 per hour (39) and >10 per hour (72). Eight mothers did not provide an answer to this question during any of the three trimesters and were excluded from the remaining analysis. Also there were 31 of 35 top CpG sites available for the F2 newborn subset.

### Gene Expression Array

At birth, IoW F2 cord blood samples were collected into PAXgene Bone Marrow RNA Tubes and RNA extracted using PAXgene RNA kits (PreAnalytiX GmbH, Switzerland). RNA integrity was verified with the Agilent 2100 Bioanalyzer system. Genome-wide mRNA expression was assessed via one color (Cy3) experiments with the Agilent (Agilent Technologies, Santa Clara, CA) SurePrint G3 Human Gene Expression 8×60k v2 microarray kits. Array content was sourced from RefSeq, Ensembl, UniGene and GenBank databases and provides full coverage of the human transcriptome in 50 599 biological features (including replicate probes and control probes). The oligos were 60mer in length and each transcript was tagged at least once and some had multiple tagging oligos for genes with documented splice variants. Data QC indices and analyses were performed with Agilent GeneSpring software. These data were then percent shift normalized and log_2_-transformed.

### Statistical Analysis

*DNA* *-* *m data*: Linear regression models, Dunnett’s multiple comparison tests and trend tests were used to assess the relationship between the frequency of vehicular traffic and DNA-m, as previously described for the subset from the F1 generation. The models were adjusted for gender and birthweight. Successful replication was defined as having the same direction of differential methylation and a *P*-value of <0.05.

Gene expression data: We calculated partial Spearman’s rank correlations between the DNA-m at 31 of 35 CpG sites and gene expression data for the associated genes while controlling for cell types (Bcell, CD4T, CD8T, gran, mono, NK and nRBC). Since cord blood includes nucleated red blood cells, we used the cell references provided by Bakulski and colleagues [118, 119].

## Supplementary Material

Supplementary Data
