# Frequency of heavy vehicle traffic and association with DNA methylation at age 18 years in a subset of the Isle of Wight birth cohort

**DOI:** 10.1093/eep/dvz003

**Published:** 2019-03-20

**Authors:** A Commodore, N Mukherjee, D Chung, E Svendsen, J Vena, J Pearce, J Roberts, S H Arshad, W Karmaus

*Environmental Epigenetics 2019*

doi: 10.1093/eep/dvy028

An error in an author name was published incorrectly and has since been corrected. The author name “H. S. Arshad” has since been fixed to correctly read as “S. H. Arshad.”

The authors apologize for this error.

