# Supplementary material for: Frequency of heavy vehicle traffic and association with DNA methylation at age 18 years in a subset of the Isle of Wight birth cohort
Source: Environ Epigenet. 2019 Jan 23;4(4):dvy028. doi: 10.1093/eep/dvy028 (PMC6343046; doi:10.1093/eep/dvy028)
Supplement: Supplementary Data [file dvy028_supp.zip › Supp table.docx]

**Supplementary Materials**

**Frequency of heavy vehicle traffic and association with DNA methylation at age 18 years in a subset of the Isle of Wight Birth Cohort**

Commodore A,^a^* Mukherjee N,^b^ Chung D,^a^ Svendsen E,^a^ Vena J,^a^ Pearce J^a^ Roberts, J^c^, Arshad H.S.^d^, and Karmaus, W. ^b^

^a^Medical University of South Carolina, Department of Public Health Sciences, Charleston, SC, USA

^b^University of Memphis, Division of Epidemiology, Biostatistics, and Environmental Health, Memphis, TN 38152, USA

^c^Medical University of South Carolina, Department of Pediatrics, Charleston, SC, USA

^d^Faculty of Medicine, University of Southampton, Southampton, UK and The David Hide Asthma and Allergy Research Centre, Isle of Wight, UK

*Corresponding Author:

Adwoa Commodore, PhD

Email: commodad@musc.edu; Telephone: +1 (843) 876-0911

Address: Department of Public Health Sciences, Medical University of South Carolina

135 Cannon St, CS303 Charleston SC 29425

Supplementary Materials

**Table S1.** Results for linear models for the top 35 CpG sites after adjusting for all confounding factors considered apriori in this study

**Table S2.** Results for linear models for CpG sites associated with the frequency of heavy vehicles passing by homes of current smokers

**Table S3.** Results for linear models for CpG sites associated with the frequency of heavy vehicles passing by homes of current nonsmokers

**Table S4.** Results for linear models for CpG sites associated with the frequency of heavy vehicles passing by homes among male subjects

**Table S5.** Results for linear models for CpG sites associated with the frequency of heavy vehicles passing by homes among female subjects

**ToppGene Results.** Detailed results for gene enrichment analysis
