# Supplementary material for: Frequency of heavy vehicle traffic and association with DNA methylation at age 18 years in a subset of the Isle of Wight birth cohort
Source: Environ Epigenet. 2019 Jan 23;4(4):dvy028. doi: 10.1093/eep/dvy028 (PMC6343046; doi:10.1093/eep/dvy028)
Supplement: Supplementary Data [file dvy028_supp.zip › Table S1.docx]

Table S1 – Results for linear models for the top 35 CpG sites after adjusting for all confounding factors considered apriori in this study

| **CpG** | **Associated Gene** | **Heavy Vehicle Frequency (ref=Never)** | **Estimate** | **Standard Error** | **P value** | **Significant covariates in model** | **Direction of Methylation** |
| --- | --- | --- | --- | --- | --- | --- | --- |
| **cg25895913 (n=329)** | ***CDH4*** |  |  |  |  |  | **↑** |
|  |  | >10 /hr | 0.16 | 0.04 | 0.0002 | Maternal Smoking; Tobacco Smoke Exposure (0-4 yrs and at 10 yrs); SES; Gender; BMI; Current smoking status; Exposure to smoke outside the home |  |
|  |  | 1-9 /hr | 0.16 | 0.04 | 0.0004 |  |  |
|  |  | 10 /day | 0.04 | 0.05 | 0.4 |  |  |
|  |  | Seldom | 0.04 | 0.04 | 0.3 |  |  |
| **cg11156891 (n=329)** | ***ANKRD65*** |  |  |  |  |  | **↓** |
|  |  | >10 /hr | -0.48 | 0.13 | 0.0002 | Maternal Smoking; Tobacco Smoke Exposure (0-4 yrs and at 10 yrs); SES; Gender; BMI; Current smoking status; Exposure to smoke outside the home |  |
|  |  | 1-9 /hr | -0.29 | 0.13 | 0.03 |  |  |
|  |  | 10 /day | -0.19 | 0.15 | 0.2 |  |  |
|  |  | Seldom | -0.12 | 0.11 | 0.3 |  |  |
| **cg12407057 (n=329)** | ***ANKRD65*** |  |  |  |  |  | **↓** |
|  |  | >10 /hr | -0.33 | 0.10 | 0.0006 | Maternal Smoking; Tobacco Smoke Exposure (0-4 yrs and at 10 yrs); SES; Gender; BMI; Current smoking status; Exposure to smoke outside the home |  |
|  |  | 1-9 /hr | -0.24 | 0.10 | 0.01 |  |  |
|  |  | 10 /day | -0.08 | 0.12 | 0.5 |  |  |
|  |  | Seldom | -0.07 | 0.09 | 0.4 |  |  |
| **cg20747739 (n=329)** | ***FAM132A*** |  |  |  |  |  | **↑** |
|  |  | >10 /hr | 0.15 | 0.04 | <.0001 | Maternal Smoking; Tobacco Smoke Exposure (0-4 yrs and at 10 yrs); SES; Gender; BMI; Current smoking status; Exposure to smoke outside the home |  |
|  |  | 1-9 /hr | 0.12 | 0.04 | 0.002 |  |  |
|  |  | 10 /day | 0.04 | 0.05 | 0.4 |  |  |
|  |  | Seldom | 0.02 | 0.03 | 0.5 |  |  |
| **cg18565510 (n=329)** | ***ACAP3*** |  |  |  |  |  | **↑** |
|  |  | >10 /hr | 0.22 | 0.05 | <.0001 | Maternal Smoking; Tobacco Smoke Exposure (0-4 yrs and at 10 yrs); SES; Gender; BMI; Current smoking status; Exposure to smoke outside the home |  |
|  |  | 1-9 /hr | 0.14 | 0.05 | 0.01 |  |  |
|  |  | 10 /day | 0.09 | 0.06 | 0.2 |  |  |
|  |  | Seldom | 0.07 | 0.05 | 0.1 |  |  |
| **cg24843003 (n=329)** | ***DAZAP1*** |  |  |  |  |  | **↑** |
|  |  | >10 /hr | 0.19 | 0.05 | 0.0001 | Maternal Smoking; Tobacco Smoke Exposure (0-4 yrs and at 10 yrs); SES; Gender; BMI; Current smoking status; Exposure to smoke outside the home |  |
|  |  | 1-9 /hr | 0.15 | 0.05 | 0.005 |  |  |
|  |  | 10 /day | 0.05 | 0.06 | 0.4 |  |  |
|  |  | Seldom | 0.09 | 0.04 | 0.06 |  |  |
| **cg15730464 (n=329)** | ***LGI2*** |  |  |  |  |  | **↓** |
|  |  | >10 /hr | 0.28 | 0.06 | <.0001 | Maternal Smoking; Tobacco Smoke Exposure (0-4 yrs and at 10 yrs); SES; Gender; BMI; Current smoking status; Exposure to smoke outside the home |  |
|  |  | 1-9 /hr | 0.19 | 0.06 | 0.004 |  |  |
|  |  | 10 /day | 0.04 | 0.07 | 0.6 |  |  |
|  |  | Seldom | 0.18 | 0.06 | 0.001 |  |  |
| **cg16196077 (n=329)** | ***RTKN2*** |  |  |  |  |  | **↓** |
|  |  | >10 /hr | -0.27 | 0.09 | 0.003 | Maternal Smoking; Tobacco Smoke Exposure (0-4 yrs and at 10 yrs); SES; Gender; BMI; Current smoking status; Exposure to smoke outside the home |  |
|  |  | 1-9 /hr | -0.25 | 0.09 | 0.007 |  |  |
|  |  | 10 /day | -0.15 | 0.11 | 0.2 |  |  |
|  |  | Seldom | 0.00 | 0.08 | 1.0 |  |  |
| **cg02707264 (n=329)** | ***MYRIP*** |  |  |  |  |  | **↓** |
|  |  | >10 /hr | -0.11 | 0.04 | 0.005 | Maternal Smoking; Tobacco Smoke Exposure (0-4 yrs and at 10 yrs); SES; Gender; BMI; Current smoking status; Exposure to smoke outside the home |  |
|  |  | 1-9 /hr | -0.02 | 0.04 | 0.6 |  |  |
|  |  | 10 /day | 0.01 | 0.05 | 0.7 |  |  |
|  |  | Seldom | 0.02 | 0.03 | 0.5 |  |  |
| **cg03476673 (n=329)** | ***CRISPLD2*** |  |  |  |  |  | **↓** |
|  |  | >10 /hr | -0.23 | 0.07 | 0.0009 | Maternal Smoking; Tobacco Smoke Exposure (0-4 yrs and at 10 yrs); SES; Gender; BMI; Current smoking status; Exposure to smoke outside the home |  |
|  |  | 1-9 /hr | -0.12 | 0.07 | 0.09 |  |  |
|  |  | 10 /day | -0.11 | 0.08 | 0.2 |  |  |
|  |  | Seldom | -0.08 | 0.06 | 0.2 |  |  |
| **cg07023532 (n=329)** | ***ACOT4*** |  |  |  |  |  | **↑** |
|  |  | >10 /hr | 0.18 | 0.06 | 0.001 | Maternal Smoking; Tobacco Smoke Exposure (0-4 yrs and at 10 yrs); SES; Gender; BMI; Current smoking status; Exposure to smoke outside the home |  |
|  |  | 1-9 /hr | 0.23 | 0.06 | <.0001 |  |  |
|  |  | 10 /day | 0.21 | 0.07 | 0.002 |  |  |
|  |  | Seldom | 0.14 | 0.05 | 0.005 |  |  |
| **cg20255272 (n=329)** | ***VWA1*** |  |  |  |  |  | **↑** |
|  |  | >10 /hr | 0.24 | 0.07 | 0.001 | Maternal Smoking; Tobacco Smoke Exposure (0-4 yrs and at 10 yrs); SES; Gender; BMI; Current smoking status; Exposure to smoke outside the home |  |
|  |  | 1-9 /hr | 0.15 | 0.08 | 0.05 |  |  |
|  |  | 10 /day | 0.10 | 0.09 | 0.3 |  |  |
|  |  | Seldom | 0.01 | 0.07 | 0.9 |  |  |
| **cg12417992 (n=329)** | ***SLC6A9*** |  |  |  |  |  | **↑** |
|  |  | >10 /hr | 0.12 | 0.04 | 0.001 | Maternal Smoking; Tobacco Smoke Exposure (0-4 yrs and at 10 yrs); SES; Gender; BMI; Current smoking status; Exposure to smoke outside the home |  |
|  |  | 1-9 /hr | 0.11 | 0.04 | 0.004 |  |  |
|  |  | 10 /day | 0.11 | 0.05 | 0.02 |  |  |
|  |  | Seldom | 0.04 | 0.03 | 0.3 |  |  |
| **cg04154465 (n=329)** | ***WNT2B*** |  |  |  |  |  | **↑** |
|  |  | >10 /hr | 0.23 | 0.07 | 0.0004 | Maternal Smoking; Tobacco Smoke Exposure (0-4 yrs and at 10 yrs); SES; Gender; BMI; Current smoking status; Exposure to smoke outside the home |  |
|  |  | 1-9 /hr | 0.19 | 0.07 | 0.004 |  |  |
|  |  | 10 /day | 0.11 | 0.08 | 0.1 |  |  |
|  |  | Seldom | 0.05 | 0.06 | 0.4 |  |  |
| **cg12813768 (n=329)** | ***SYCP1*** |  |  |  |  |  | **↓** |
|  |  | >10 /hr | -0.37 | 0.09 | <.0001 | Maternal Smoking; Tobacco Smoke Exposure (0-4 yrs and at 10 yrs); SES; Gender; BMI; Current smoking status; Exposure to smoke outside the home |  |
|  |  | 1-9 /hr | -0.19 | 0.09 | 0.04 |  |  |
|  |  | 10 /day | -0.09 | 0.11 | 0.4 |  |  |
|  |  | Seldom | -0.08 | 0.08 | 0.3 |  |  |
| **cg14162906 (n=329)** | ***TMEM222*** |  |  |  |  |  | **↑** |
|  |  | >10 /hr | 0.14 | 0.04 | 0.0005 | Maternal Smoking; Tobacco Smoke Exposure (0-4 yrs and at 10 yrs); SES; Gender; BMI; Current smoking status; Exposure to smoke outside the home |  |
|  |  | 1-9 /hr | 0.15 | 0.04 | 0.0005 |  |  |
|  |  | 10 /day | 0.15 | 0.05 | 0.003 |  |  |
|  |  | Seldom | 0.07 | 0.04 | 0.08 |  |  |
| **cg24361098 (n=329)** | ***BCL11A*** |  |  |  |  |  | **↑** |
|  |  | >10 /hr | 0.23 | 0.06 | 0.0002 | Maternal Smoking; Tobacco Smoke Exposure (0-4 yrs and at 10 yrs); SES; Gender; BMI; Current smoking status; Exposure to smoke outside the home |  |
|  |  | 1-9 /hr | 0.15 | 0.06 | 0.02 |  |  |
|  |  | 10 /day | 0.13 | 0.07 | 0.09 |  |  |
|  |  | Seldom | 0.13 | 0.06 | 0.02 |  |  |
| **cg16147794 (n=329)** | ***SLC16A10*** |  |  |  |  |  | **↓** |
|  |  | >10 /hr | -0.19 | 0.07 | 0.006 | Maternal Smoking; Tobacco Smoke Exposure (0-4 yrs and at 10 yrs); SES; Gender; BMI; Current smoking status; Exposure to smoke outside the home |  |
|  |  | 1-9 /hr | -0.07 | 0.07 | 0.3 |  |  |
|  |  | 10 /day | -0.13 | 0.08 | 0.1 |  |  |
|  |  | Seldom | 0.06 | 0.06 | 0.4 |  |  |
| **cg16668397 (n=329)** | ***JPH3*** |  |  |  |  |  | **↑** |
|  |  | >10 /hr | 0.11 | 0.04 | 0.01 | Maternal Smoking; Tobacco Smoke Exposure (0-4 yrs and at 10 yrs); SES; Gender; BMI; Current smoking status; Exposure to smoke outside the home |  |
|  |  | 1-9 /hr | 0.12 | 0.05 | 0.007 |  |  |
|  |  | 10 /day | 0.07 | 0.05 | 0.2 |  |  |
|  |  | Seldom | 0.02 | 0.04 | 0.6 |  |  |
| **cg26419883 (n=329)** | ***TRPM5*** |  |  |  |  |  | **↑** |
|  |  | >10 /hr | 0.18 | 0.04 | <.0001 | Maternal Smoking; Tobacco Smoke Exposure (0-4 yrs and at 10 yrs); SES; Gender; BMI; Current smoking status; Exposure to smoke outside the home |  |
|  |  | 1-9 /hr | 0.05 | 0.05 | 0.3 |  |  |
|  |  | 10 /day | 0.06 | 0.05 | 0.2 |  |  |
|  |  | Seldom | 0.03 | 0.04 | 0.4 |  |  |
| **cg21775675 (n=329)** | ***TMEM161B*** |  |  |  |  |  | **↓** |
|  |  | >10 /hr | -0.17 | 0.05 | 0.001 | Maternal Smoking; Tobacco Smoke Exposure (0-4 yrs and at 10 yrs); SES; Gender; BMI; Current smoking status; Exposure to smoke outside the home |  |
|  |  | 1-9 /hr | -0.05 | 0.05 | 0.3 |  |  |
|  |  | 10 /day | -0.09 | 0.06 | 0.2 |  |  |
|  |  | Seldom | 0.00 | 0.05 | 1.0 |  |  |
| **cg04794690 (n=329)** | ***PADI3*** |  |  |  |  |  | **↑** |
|  |  | >10 /hr | 0.16 | 0.05 | 0.004 | Maternal Smoking; Tobacco Smoke Exposure (0-4 yrs and at 10 yrs); SES; Gender; BMI; Current smoking status; Exposure to smoke outside the home |  |
|  |  | 1-9 /hr | 0.20 | 0.05 | 0.0003 |  |  |
|  |  | 10 /day | 0.07 | 0.06 | 0.3 |  |  |
|  |  | Seldom | 0.12 | 0.05 | 0.02 |  |  |
| **cg06942649 (n=329)** | ***FBXO25*** |  |  |  |  |  | **↑** |
|  |  | >10 /hr | 0.23 | 0.09 | 0.01 | Maternal Smoking; Tobacco Smoke Exposure (0-4 yrs and at 10 yrs); SES; Gender; BMI; Current smoking status; Exposure to smoke outside the home |  |
|  |  | 1-9 /hr | 0.23 | 0.09 | 0.01 |  |  |
|  |  | 10 /day | 0.20 | 0.11 | 0.06 |  |  |
|  |  | Seldom | 0.06 | 0.08 | 0.4 |  |  |
| **cg18459806 (n=329)** | ***NIN*** |  |  |  |  |  | **↓** |
|  |  | >10 /hr | -0.16 | 0.04 | <.0001 | Maternal Smoking; Tobacco Smoke Exposure (0-4 yrs and at 10 yrs); SES; Gender; BMI; Current smoking status; Exposure to smoke outside the home |  |
|  |  | 1-9 /hr | -0.07 | 0.04 | 0.07 |  |  |
|  |  | 10 /day | -0.10 | 0.05 | 0.04 |  |  |
|  |  | Seldom | -0.03 | 0.04 | 0.4 |  |  |
| **cg20631351 (n=329)** | ***PALM*** |  |  |  |  |  | **↑** |
|  |  | >10 /hr | 0.12 | 0.04 | 0.004 | Maternal Smoking; Tobacco Smoke Exposure (0-4 yrs and at 10 yrs); SES; Gender; BMI; Current smoking status; Exposure to smoke outside the home |  |
|  |  | 1-9 /hr | 0.08 | 0.04 | 0.05 |  |  |
|  |  | 10 /day | 0.06 | 0.05 | 0.2 |  |  |
|  |  | Seldom | 0.04 | 0.04 | 0.3 |  |  |
| **cg00347824 (n=329)** | ***NSMAF*** |  |  |  |  |  | **↑** |
|  |  | >10 /hr | 0.21 | 0.05 | 0.0002 | Maternal Smoking; Tobacco Smoke Exposure (0-4 yrs and at 10 yrs); SES; Gender; BMI; Current smoking status; Exposure to smoke outside the home |  |
|  |  | 1-9 /hr | 0.10 | 0.06 | 0.08 |  |  |
|  |  | 10 /day | 0.12 | 0.07 | 0.06 |  |  |
|  |  | Seldom | 0.06 | 0.05 | 0.3 |  |  |
| **cg17053854 (n=329)** | ***SEPT9*** |  |  |  |  |  | **↑** |
|  |  | >10 /hr | 0.09 | 0.03 | 0.002 | Maternal Smoking; Tobacco Smoke Exposure (0-4 yrs and at 10 yrs); SES; Gender; BMI; Current smoking status; Exposure to smoke outside the home |  |
|  |  | 1-9 /hr | 0.09 | 0.03 | 0.004 |  |  |
|  |  | 10 /day | 0.03 | 0.04 | 0.4 |  |  |
|  |  | Seldom | 0.02 | 0.03 | 0.5 |  |  |
| **cg25324786 (n=329)** | ***RASA3*** |  |  |  |  |  | **↑** |
|  |  | >10 /hr | 0.13 | 0.05 | 0.01 | Maternal Smoking; Tobacco Smoke Exposure (0-4 yrs and at 10 yrs); SES; Gender; BMI; Current smoking status; Exposure to smoke outside the home |  |
|  |  | 1-9 /hr | 0.07 | 0.05 | 0.2 |  |  |
|  |  | 10 /day | -0.04 | 0.06 | 0.5 |  |  |
|  |  | Seldom | -0.04 | 0.05 | 0.4 |  |  |
| **cg26720961 (n=329)** | ***TSNARE1*** |  |  |  |  |  | **↑** |
|  |  | >10 /hr | 0.12 | 0.06 | 0.05 | Maternal Smoking; Tobacco Smoke Exposure (0-4 yrs and at 10 yrs); SES; Gender; BMI; Current smoking status; Exposure to smoke outside the home |  |
|  |  | 1-9 /hr | 0.02 | 0.06 | 0.8 |  |  |
|  |  | 10 /day | -0.01 | 0.08 | 0.8 |  |  |
|  |  | Seldom | -0.10 | 0.06 | 0.06 |  |  |
| **cg05575058 (n=329)** | ***FAM164A*** |  |  |  |  |  | **↓** |
|  |  | >10 /hr | -0.16 | 0.05 | 0.0008 | Maternal Smoking; Tobacco Smoke Exposure (0-4 yrs and at 10 yrs); SES; Gender; BMI; Current smoking status; Exposure to smoke outside the home |  |
|  |  | 1-9 /hr | -0.08 | 0.05 | 0.09 |  |  |
|  |  | 10 /day | -0.10 | 0.06 | 0.1 |  |  |
|  |  | Seldom | -0.06 | 0.04 | 0.2 |  |  |
| **cg15742605 (n=329)** | ***SAMD11*** |  |  |  |  |  | **↑** |
|  |  | >10 /hr | 0.19 | 0.05 | 0.0003 | Maternal Smoking; Tobacco Smoke Exposure (0-4 yrs and at 10 yrs); SES; Gender; BMI; Current smoking status; Exposure to smoke outside the home |  |
|  |  | 1-9 /hr | 0.08 | 0.05 | 0.1 |  |  |
|  |  | 10 /day | 0.04 | 0.06 | 0.5 |  |  |
|  |  | Seldom | -0.01 | 0.05 | 0.9 |  |  |
| **cg26185508 (n=329)** | ***CDCP2*** |  |  |  |  |  | **↑** |
|  |  | >10 /hr | 0.16 | 0.05 | 0.004 | Maternal Smoking; Tobacco Smoke Exposure (0-4 yrs and at 10 yrs); SES; Gender; BMI; Current smoking status; Exposure to smoke outside the home |  |
|  |  | 1-9 /hr | 0.17 | 0.06 | 0.002 |  |  |
|  |  | 10 /day | 0.18 | 0.06 | 0.007 |  |  |
|  |  | Seldom | 0.08 | 0.05 | 0.1 |  |  |
| **cg02378006 (n=329)** | ***UNC5B*** |  |  |  |  |  | **↑** |
|  |  | >10 /hr | 0.19 | 0.06 | 0.001 | Maternal Smoking; Tobacco Smoke Exposure (0-4 yrs and at 10 yrs); SES; Gender; BMI; Current smoking status; Exposure to smoke outside the home |  |
|  |  | 1-9 /hr | 0.07 | 0.06 | 0.2 |  |  |
|  |  | 10 /day | 0.00 | 0.07 | 1.0 |  |  |
|  |  | Seldom | 0.00 | 0.05 | 1.0 |  |  |
| **cg08462127 (n=329)** | ***MYOM2*** |  |  |  |  |  | **↑** |
|  |  | >10 /hr | 0.13 | 0.05 | 0.006 | Maternal Smoking; Tobacco Smoke Exposure (0-4 yrs and at 10 yrs); SES; Gender; BMI; Current smoking status; Exposure to smoke outside the home |  |
|  |  | 1-9 /hr | 0.04 | 0.05 | 0.4 |  |  |
|  |  | 10 /day | 0.11 | 0.06 | 0.06 |  |  |
|  |  | Seldom | -0.02 | 0.04 | 0.7 |  |  |
| **cg11017318 (n=329)** | ***SYT16*** |  |  |  |  |  |  |
|  |  | >10 /hr | -0.06 | 0.04 | 0.1 | Maternal Smoking; Tobacco Smoke Exposure (0-4 yrs and at 10 yrs); SES; Gender; BMI; Current smoking status; Exposure to smoke outside the home |  |
|  |  | 1-9 /hr | -0.05 | 0.04 | 0.2 |  |  |
|  |  | 10 /day | -0.08 | 0.05 | 0.1 |  |  |
|  |  | Seldom | 0.00 | 0.04 | 1.0 |  |  |
