# Supplementary material for: Frequency of heavy vehicle traffic and association with DNA methylation at age 18 years in a subset of the Isle of Wight birth cohort
Source: Environ Epigenet. 2019 Jan 23;4(4):dvy028. doi: 10.1093/eep/dvy028 (PMC6343046; doi:10.1093/eep/dvy028)
Supplement: Supplementary Data [file dvy028_supp.zip › Table S2.docx]

| Table S2. Results for linear models for CpG sites associated with the frequency of heavy vehicles passing by homes of current smokers | | | | | | | | | |
| --- | --- | --- | --- | --- | --- | --- | --- | --- | --- |
| **CpG** | **Associated Gene** | **Heavy Vehicle Frequency (ref=Never)** | **Estimate** | **Standard Error** | **P value** | **Significant covariates in final model** | **Dunnett's test (LSMEAN=Never)** | **Linear trend test (F value, df=1)** *p value* | **Direction of Methylation** |
| cg25895913 (n=88) | ***CDH4*** |  |  |  |  |  |  |  | **↑** |
|  |  | >10 /hr | 0.23 | 0.08 | 0.005 | Tobacco Smoke Exposure (at 10 yrs); Gender; Age subject started smoking | * | 0.0005 |  |
|  |  | 1-9 /hr | 0.18 | 0.09 | 0.04 |  |  |  |  |
|  |  | 10 /day | -0.01 | 0.09 | 0.9 |  |  |  |  |
|  |  | Seldom | 0.10 | 0.08 | 0.2 |  |  |  |  |
| cg20747739 (n=94) | ***FAM132A*** |  |  |  |  |  |  |  | **↑** |
|  |  | >10 /hr | 0.11 | 0.07 | 0.09 | Gender; BMI; Age subject started smoking | * | 0.006 |  |
|  |  | 1-9 /hr | 0.21 | 0.07 | 0.006 |  |  |  |  |
|  |  | 10 /day | 0.07 | 0.08 | 0.4 |  |  |  |  |
|  |  | Seldom | -0.04 | 0.06 | 0.6 |  |  |  |  |
| cg18565510 (n=94) | ***ACAP3*** |  |  |  |  |  |  |  | **↑** |
|  |  | >10 /hr | 0.24 | 0.09 | 0.01 | Gender; BMI; Age subject started smoking | * | 0.01 |  |
|  |  | 1-9 /hr | 0.09 | 0.10 | 0.4 |  |  |  |  |
|  |  | 10 /day | 0.09 | 0.11 | 0.5 |  |  |  |  |
|  |  | Seldom | 0.07 | 0.09 | 0.4 |  |  |  |  |
| cg15730464 (n=87) | ***LGI2*** |  |  |  |  |  |  |  | **↓** |
|  |  | >10 /hr | 0.35 | 0.11 | 0.002 | Tobacco Smoke Exposure ( at 10 yrs); SES; Gender; Age subject started smoking | ** | <.0001 |  |
|  |  | 1-9 /hr | 0.24 | 0.12 | 0.04 |  |  |  |  |
|  |  | 10 /day | -0.22 | 0.13 | 0.1 |  |  |  |  |
|  |  | Seldom | 0.20 | 0.10 | 0.06 |  |  |  |  |
| cg16196077 (n=82) | ***RTKN2*** |  |  |  |  |  |  |  | **↓** |
|  |  | >10 /hr | -0.50 | 0.18 | 0.007 | Maternal Smoking; Tobacco Smoke Exposure (0-4 yrs and at 10 yrs); SES; Gender; Current smoking status; Exposure to smoke outside the home; Age subject started smoking | * | 0.0007 |  |
|  |  | 1-9 /hr | -0.27 | 0.21 | 0.2 |  |  |  |  |
|  |  | 10 /day | -0.14 | 0.22 | 0.5 |  |  |  |  |
|  |  | Seldom | 0.00 | 0.19 | 1.0 |  |  |  |  |
| cg07023532 (n=94) | ***ACOT4*** |  |  |  |  |  |  |  | **↑** |
|  |  | >10 /hr | 0.39 | 0.10 | 0.0003 | Gender; BMI; Age subject started smoking | ** | 0.0008 |  |
|  |  | 1-9 /hr | 0.32 | 0.11 | 0.0059 |  | * |  |  |
|  |  | 10 /day | 0.24 | 0.13 | 0.0546 |  |  |  |  |
|  |  | Seldom | 0.18 | 0.10 | 0.0812 |  |  |  |  |
| cg12813768 (n=82) | ***SYCP1*** |  |  |  |  |  |  |  | **↓** |
|  |  | >10 /hr | -0.54 | 0.19 | 0.006 | Maternal Smoking; Tobacco Smoke Exposure (0-4 yrs and at 10 yrs); SES; Gender; Exposure to smoke outside the home; Age subject started smoking | * | 0.002 |  |
|  |  | 1-9 /hr | -0.32 | 0.22 | 0.1458 |  |  |  |  |
|  |  | 10 /day | -0.08 | 0.23 | 0.7181 |  |  |  |  |
|  |  | Seldom | -0.20 | 0.20 | 0.3012 |  |  |  |  |
| cg16147794 (n=81) | ***SLC16A10*** |  |  |  |  |  |  |  | **↓** |
|  |  | >10 /hr | -0.39 | 0.13 | 0.0035 | Maternal Smoking; Tobacco Smoke Exposure (0-4 yrs and at 10 yrs); SES; Gender; BMI; Exposure to smoke outside the home; Age subject started smoking | * | 0.01 |  |
|  |  | 1-9 /hr | -0.20 | 0.15 | 0.1813 |  |  |  |  |
|  |  | 10 /day | -0.36 | 0.16 | 0.0243 |  | 0 |  |  |
|  |  | Seldom | -0.25 | 0.13 | 0.0616 |  |  |  |  |
| cg26419883 (n=94) | ***TRPM5*** |  |  |  |  |  |  |  | **↑** |
|  |  | >10 /hr | 0.20 | 0.09 | 0.0196 | BMI; Gender; Age subject started smoking | 0 | 0.02 |  |
|  |  | 1-9 /hr | 0.10 | 0.10 | 0.2807 |  |  |  |  |
|  |  | 10 /day | 0.05 | 0.10 | 0.6036 |  |  |  |  |
|  |  | Seldom | 0.10 | 0.08 | 0.2262 |  |  |  |  |
| cg21775675 (n=81) | ***TMEM161B*** |  |  |  |  |  |  |  | **↓** |
|  |  | >10 /hr | -0.37 | 0.12 | 0.002 | Maternal Smoking; Tobacco Smoke Exposure (0-4 yrs and at 10 yrs); SES; Gender; BMI; Exposure to smoke outside the home; Age subject started smoking | ** | 0.006 |  |
|  |  | 1-9 /hr | -0.03 | 0.13 | 0.8358 |  |  |  |  |
|  |  | 10 /day | -0.21 | 0.14 | 0.1466 |  |  |  |  |
|  |  | Seldom | -0.07 | 0.12 | 0.551 |  |  |  |  |
| cg06942649 (n=94) | ***FBXO25*** |  |  |  |  |  |  |  | **↑** |
|  |  | >10 /hr | 0.52 | 0.17 | 0.0024 | BMI; Gender; Age subject started smoking | ** | 0.005 |  |
|  |  | 1-9 /hr | 0.59 | 0.18 | 0.002 |  | ** |  |  |
|  |  | 10 /day | 0.40 | 0.20 | 0.0524 |  |  |  |  |
|  |  | Seldom | 0.27 | 0.16 | 0.1002 |  |  |  |  |
| cg18459806 (n=81) | ***NIN*** |  |  |  |  |  |  |  | **↓** |
|  |  | >10 /hr | -0.20 | 0.07 | 0.0101 | Maternal Smoking; Tobacco Smoke Exposure (0-4 yrs and at 10 yrs); SES; Gender; BMI; Exposure to smoke outside the home; Age subject started smoking | * | 0.009 |  |
|  |  | 1-9 /hr | -0.22 | 0.08 | 0.0125 |  | ***** |  |  |
|  |  | 10 /day | -0.14 | 0.09 | 0.1363 |  |  |  |  |
|  |  | Seldom | -0.10 | 0.08 | 0.2004 |  |  |  |  |

*** p<0.001

** p<0.01

* p<0.05

◌ p<0.1

Once the Dunnett’ tests provided statistical evidence of differences in marginal means of the heavy vehicular traffic frequency, a second test for trend is performed to assess a ‘dose-response’ relationship.
