# Supplementary material for: Frequency of heavy vehicle traffic and association with DNA methylation at age 18 years in a subset of the Isle of Wight birth cohort
Source: Environ Epigenet. 2019 Jan 23;4(4):dvy028. doi: 10.1093/eep/dvy028 (PMC6343046; doi:10.1093/eep/dvy028)
Supplement: Supplementary Data [file dvy028_supp.zip › Table S3.docx]

| Table S3. Results for linear models for CpG sites associated with the frequency of heavy vehicles passing by homes of current nonsmokers | | | | | | | | | |
| --- | --- | --- | --- | --- | --- | --- | --- | --- | --- |
| **CpG** | **Associated Gene** | **Heavy Vehicle Frequency (ref=Never)** | **Estimate** | **Standard Error** | **P value** | **Significant covariates in final model** | **Dunnett's test (LSMEAN=Never)** | **Linear trend test (F value, df=1)** *p value* | **Direction of Methylation** |
| cg25895913 (n=264) | ***CDH4*** |  |  |  |  |  |  |  | **↑** |
|  |  | >10 /hr | 0.13 | 0.05 | 0.008 | Tobacco Smoke Exposure (at 10 yrs); Gender | * | 0.005 |  |
|  |  | 1-9 /hr | 0.16 | 0.05 | 0.0006 |  | ** |  |  |
|  |  | 10 /day | 0.09 | 0.06 | 0.1 |  |  |  |  |
|  |  | Seldom | 0.03 | 0.04 | 0.4 |  |  |  |  |
| cg11156891 (n=248) | ***ANKRD65*** |  |  |  |  |  |  |  | **↓** |
|  |  | >10 /hr | -0.47 | 0.14 | 0.0009 | Maternal Smoking; Tobacco Smoke Exposure (0-4 yrs and at 10 yrs); SES; Gender; BMI; ; Exposure to smoke outside the home | ** | 0.01 |  |
|  |  | 1-9 /hr | -0.31 | 0.14 | 0.03 |  | 0 |  |  |
|  |  | 10 /day | -0.44 | 0.17 | 0.01 |  | * |  |  |
|  |  | Seldom | -0.14 | 0.12 | 0.3 |  |  |  |  |
| cg12407057 (n=248) | ***ANKRD65*** |  |  |  |  |  |  |  | **↓** |
|  |  | >10 /hr | -0.32 | 0.11 | 0.003 | Maternal Smoking; Tobacco Smoke Exposure (0-4 yrs and at 10 yrs); SES; Gender; BMI; Exposure to smoke outside the home | ** | 0.01 |  |
|  |  | 1-9 /hr | -0.25 | 0.10 | 0.02 |  | 0 |  |  |
|  |  | 10 /day | -0.25 | 0.13 | 0.05 |  |  |  |  |
|  |  | Seldom | -0.09 | 0.09 | 0.3 |  |  |  |  |
| cg20747739 (n=264) | ***FAM132A*** |  |  |  |  |  |  |  | **↑** |
|  |  | >10 /hr | 0.15 | 0.04 | 0.0007 | Gender; BMI | ** | 0.0003 |  |
|  |  | 1-9 /hr | 0.08 | 0.04 | 0.05 |  |  |  |  |
|  |  | 10 /day | 0.02 | 0.05 | 0.7 |  |  |  |  |
|  |  | Seldom | 0.05 | 0.04 | 0.2 |  |  |  |  |
| cg18565510 (n=264) | ***ACAP3*** |  |  |  |  |  |  |  | **↑** |
|  |  | >10 /hr | 0.19 | 0.06 | 0.002 | Gender; BMI | ** | 0.002 |  |
|  |  | 1-9 /hr | 0.16 | 0.06 | 0.008 |  | * |  |  |
|  |  | 10 /day | 0.08 | 0.07 | 0.3 |  |  |  |  |
|  |  | Seldom | 0.06 | 0.05 | 0.2 |  |  |  |  |
| cg24843003 (n=270) | ***DAZAP1*** |  |  |  |  |  |  |  | **↑** |
|  |  | >10 /hr | 0.20 | 0.05 | 0.0004 | Gender | ** | 0.001 |  |
|  |  | 1-9 /hr | 0.15 | 0.05 | 0.007 |  | * |  |  |
|  |  | 10 /day | 0.09 | 0.07 | 0.2 |  |  |  |  |
|  |  | Seldom | 0.08 | 0.05 | 0.10 |  |  |  |  |
| cg15730464 (n=258) | ***LGI2*** |  |  |  |  |  |  |  | **↓** |
|  |  | >10 /hr | 0.28 | 0.07 | 0.0002 | Tobacco Smoke Exposure ( at 10 yrs); SES; Gender | ** | 0.004 |  |
|  |  | 1-9 /hr | 0.18 | 0.07 | 0.01 |  | 0 |  |  |
|  |  | 10 /day | 0.15 | 0.09 | 0.09 |  |  |  |  |
|  |  | Seldom | 0.17 | 0.06 | 0.01 |  | * |  |  |
| cg03476673 (n=248) | ***CRISPLD2*** |  |  |  |  |  |  |  | **↓** |
|  |  | >10 /hr | -0.23 | 0.08 | 0.004 | Maternal Smoking; Tobacco Smoke Exposure (0-4 yrs and at 10 yrs); SES; Gender; BMI; Exposure to smoke outside the home | * | 0.01 |  |
|  |  | 1-9 /hr | -0.14 | 0.08 | 0.07 |  |  |  |  |
|  |  | 10 /day | -0.15 | 0.09 | 0.1 |  |  |  |  |
|  |  | Seldom | -0.07 | 0.07 | 0.3 |  |  |  |  |
| cg20255272 (n=258) | ***VWA1*** |  |  |  |  |  |  |  | **↑** |
|  |  | >10 /hr | 0.21 | 0.08 | 0.01 | Tobacco Smoke Exposure (0-4 yrs only); Gender; BMI; SES; | * | 0.03 |  |
|  |  | 1-9 /hr | 0.10 | 0.08 | 0.2 |  |  |  |  |
|  |  | 10 /day | 0.15 | 0.10 | 0.1 |  |  |  |  |
|  |  | Seldom | 0.04 | 0.07 | 0.6 |  |  |  |  |
| cg12417992 (n=264) | ***SLC6A9*** |  |  |  |  |  |  |  | **↑** |
|  |  | >10 /hr | 0.13 | 0.04 | 0.004 | Maternal Smoking; Tobacco Smoke Exposure (0-4 yrs only); SES; Gender | * | 0.01 |  |
|  |  | 1-9 /hr | 0.11 | 0.04 | 0.02 |  | 0 |  |  |
|  |  | 10 /day | 0.12 | 0.05 | 0.03 |  |  |  |  |
|  |  | Seldom | 0.03 | 0.04 | 0.5 |  |  |  |  |
| cg04154465 (n=258) | ***WNT2B*** |  |  |  |  |  |  |  | **↑** |
|  |  | >10 /hr | 0.28 | 0.07 | 0.0001 | SES; Gender; BMI | *** | <.0001 |  |
|  |  | 1-9 /hr | 0.22 | 0.07 | 0.002 |  | ** |  |  |
|  |  | 10 /day | 0.09 | 0.09 | 0.3 |  |  |  |  |
|  |  | Seldom | 0.08 | 0.06 | 0.2 |  |  |  |  |
| cg12813768 (n=254) | ***SYCP1*** |  |  |  |  |  |  |  | **↓** |
|  |  | >10 /hr | -0.28 | 0.10 | 0.007 | Maternal Smoking; Tobacco Smoke Exposure (0-4 yrs and at 10 yrs); SES; Gender; Exposure to smoke outside the home | * | 0.009 |  |
|  |  | 1-9 /hr | -0.16 | 0.10 | 0.1 |  |  |  |  |
|  |  | 10 /day | -0.11 | 0.13 | 0.4 |  |  |  |  |
|  |  | Seldom | -0.06 | 0.09 | 0.5 |  |  |  |  |
| cg14162906 (n=264) | ***TMEM222*** |  |  |  |  |  |  |  | **↑** |
|  |  | >10 /hr | 0.14 | 0.05 | 0.003 | BMI; Gender | * | 0.03 |  |
|  |  | 1-9 /hr | 0.15 | 0.05 | 0.002 |  | ** |  |  |
|  |  | 10 /day | 0.18 | 0.06 | 0.003 |  | ****** |  |  |
|  |  | Seldom | 0.04 | 0.04 | 0.3 |  |  |  |  |
| cg24361098 (n=264) | ***BCL11A*** |  |  |  |  |  |  |  | **↑** |
|  |  | >10 /hr | 0.25 | 0.07 | 0.0004 | BMI; Gender | ** | 0.004 |  |
|  |  | 1-9 /hr | 0.18 | 0.07 | 0.009 |  | ***** |  |  |
|  |  | 10 /day | 0.16 | 0.09 | 0.07 |  |  |  |  |
|  |  | Seldom | 0.11 | 0.06 | 0.07 |  |  |  |  |
| cg16668397 (n=264) | ***JPH3*** |  |  |  |  |  |  |  | **↑** |
|  |  | >10 /hr | 0.13 | 0.05 | 0.008 | BMI; Gender | * | 0.01 |  |
|  |  | 1-9 /hr | 0.13 | 0.05 | 0.007 |  | * |  |  |
|  |  | 10 /day | 0.11 | 0.06 | 0.06 |  |  |  |  |
|  |  | Seldom | 0.03 | 0.04 | 0.5 |  |  |  |  |
| cg26419883 (n=264) | ***TRPM5*** |  |  |  |  |  |  |  | **↑** |
|  |  | >10 /hr | 0.15 | 0.05 | 0.003 | BMI; Gender | * | 0.003 |  |
|  |  | 1-9 /hr | 0.05 | 0.05 | 0.3 |  |  |  |  |
|  |  | 10 /day | 0.06 | 0.06 | 0.4 |  |  |  |  |
|  |  | Seldom | 0.01 | 0.04 | 0.9 |  |  |  |  |
| cg04794690 (n=264) | ***PADI3*** |  |  |  |  |  |  |  | **↑** |
|  |  | >10 /hr | 0.19 | 0.06 | 0.002 | BMI; Gender | ** | 0.001 |  |
|  |  | 1-9 /hr | 0.22 | 0.06 | 0.0003 |  | ** |  |  |
|  |  | 10 /day | 0.05 | 0.07 | 0.5 |  |  |  |  |
|  |  | Seldom | 0.10 | 0.05 | 0.06 |  |  |  |  |
| cg18459806 (n=248) | ***NIN*** |  |  |  |  |  |  |  | **↓** |
|  |  | >10 /hr | -0.15 | 0.05 | 0.002 | Maternal Smoking; Tobacco Smoke Exposure (0-4 yrs and at 10 yrs); SES; Gender; BMI; Exposure to smoke outside the home | ** | 0.007 |  |
|  |  | 1-9 /hr | -0.03 | 0.05 | 0.5 |  |  |  |  |
|  |  | 10 /day | -0.09 | 0.06 | 0.1 |  |  |  |  |
|  |  | Seldom | -0.01 | 0.04 | 0.8 |  |  |  |  |
| cg20631351 (n=264) | ***PALM*** |  |  |  |  |  |  |  | **↑** |
|  |  | >10 /hr | 0.11 | 0.04 | 0.01 | BMI; Gender | 0 | 0.01 |  |
|  |  | 1-9 /hr | 0.11 | 0.04 | 0.01 |  | ***** |  |  |
|  |  | 10 /day | 0.06 | 0.05 | 0.3 |  |  |  |  |
|  |  | Seldom | 0.04 | 0.04 | 0.3 |  |  |  |  |
| cg00347824 (n=264) | ***NSMAF*** |  |  |  |  |  |  |  | **↑** |
|  |  | >10 /hr | 0.22 | 0.06 | 0.0005 | BMI; Gender | ** | 0.007 |  |
|  |  | 1-9 /hr | 0.10 | 0.06 | 0.1 |  |  |  |  |
|  |  | 10 /day | 0.15 | 0.08 | 0.06 |  |  |  |  |
|  |  | Seldom | 0.08 | 0.06 | 0.1 |  |  |  |  |
| cg17053854 (n=258) | ***SEPT9*** |  |  |  |  |  |  |  | **↑** |
|  |  | >10 /hr | 0.09 | 0.03 | 0.01 | Tobacco Smoke Exposure (at 10 yrs ); BMI; Gender | * | 0.003 |  |
|  |  | 1-9 /hr | 0.09 | 0.03 | 0.007 |  | * |  |  |
|  |  | 10 /day | 0.05 | 0.04 | 0.3 |  |  |  |  |
|  |  | Seldom | 0.00 | 0.03 | 1.0 |  |  |  |  |
| cg26720961 (n=254) | ***TSNARE1*** |  |  |  |  |  |  |  | **↑** |
|  |  | >10 /hr | 0.16 | 0.07 | 0.03 | Maternal Smoking; Tobacco Smoke Exposure (0-4 yrs and at 10 yrs); SES; Gender; Exposure to smoke outside the home | 0 | 0.002 |  |
|  |  | 1-9 /hr | 0.04 | 0.07 | 0.6 |  |  |  |  |
|  |  | 10 /day | 0.00 | 0.09 | 1.0 |  |  |  |  |
|  |  | Seldom | -0.10 | 0.06 | 0.1 |  |  |  |  |
| cg05575058 (n=248) | ***FAM164A*** |  |  |  |  |  |  |  | **↓** |
|  |  | >10 /hr | -0.19 | 0.06 | 0.0008 | Maternal Smoking; Tobacco Smoke Exposure (0-4 yrs and at 10 yrs); SES; Gender; BMI; Exposure to smoke outside the home | ** | 0.003 |  |
|  |  | 1-9 /hr | -0.11 | 0.05 | 0.05 |  |  |  |  |
|  |  | 10 /day | -0.10 | 0.07 | 0.1 |  |  |  |  |
|  |  | Seldom | -0.05 | 0.05 | 0.3 |  |  |  |  |
| cg15742605 (n=248) | ***SAMD11*** |  |  |  |  |  |  |  | **↑** |
|  |  | >10 /hr | 0.16 | 0.06 | 0.007 | Maternal Smoking; Tobacco Smoke Exposure (0-4 yrs and at 10 yrs); SES; Gender; BMI; Exposure to smoke outside the home | * | 0.0003 |  |
|  |  | 1-9 /hr | 0.05 | 0.06 | 0.4 |  |  |  |  |
|  |  | 10 /day | -0.01 | 0.07 | 0.9 |  |  |  |  |
|  |  | Seldom | -0.05 | 0.05 | 0.3 |  |  |  |  |
| cg26185508 (n=264) | ***CDCP2*** |  |  |  |  |  |  |  | **↑** |
|  |  | >10 /hr | 0.13 | 0.06 | 0.03 | BMI; Gender | * | 0.08 |  |
|  |  | 1-9 /hr | 0.17 | 0.06 | 0.004 |  |  |  |  |
|  |  | 10 /day | 0.12 | 0.07 | 0.1 |  |  |  |  |
|  |  | Seldom | 0.07 | 0.05 | 0.1 |  |  |  |  |
| cg02378006 (n=248) | ***UNC5B*** |  |  |  |  |  |  |  | **↑** |
|  |  | >10 /hr | 0.17 | 0.07 | 0.02 | Maternal Smoking; Tobacco Smoke Exposure (0-4 yrs and at 10 yrs); SES; Gender; BMI; Exposure to smoke outside the home | 0 | 0.002 |  |
|  |  | 1-9 /hr | 0.07 | 0.07 | 0.3 |  |  |  |  |
|  |  | 10 /day | -0.01 | 0.09 | 0.9 |  |  |  |  |
|  |  | Seldom | -0.02 | 0.06 | 0.8 |  |  |  |  |
|  |  |  |  |  |  |  |  |  |  |

*** p<0.001

** p<0.01

* p<0.05

◌ p<0.1

Once the Dunnett’ tests provided statistical evidence of differences in marginal means of the heavy vehicular traffic frequency, a second test for trend is performed to assess a ‘dose-response’ relationship.
