# Supplementary material for: Frequency of heavy vehicle traffic and association with DNA methylation at age 18 years in a subset of the Isle of Wight birth cohort
Source: Environ Epigenet. 2019 Jan 23;4(4):dvy028. doi: 10.1093/eep/dvy028 (PMC6343046; doi:10.1093/eep/dvy028)
Supplement: Supplementary Data [file dvy028_supp.zip › Table S4.docx]

| Table S4. Results for linear models for CpG sites associated with the frequency of heavy vehicles passing by homes among male subjects | | | | | | | | | |
| --- | --- | --- | --- | --- | --- | --- | --- | --- | --- |
| **CpG** | **Associated Gene** | **Heavy Vehicle Frequency (ref=Never)** | **Estimate** | **Standard Error** | **P value** | **Significant covariates in final model** | **Dunnett's test (LSMEAN=Never)** | **Linear trend test (F value, df=1)** *p value* | **Direction of Methylation** |
| cg18565510 (n=122) | **ACAP3** |  |  |  |  |  |  |  | **↑** |
|  |  | >10 /hr | 0.15 | 0.06 | 0.02 | Gender; BMI | 0 | 3.9 |  |
|  |  | 1-9 /hr | 0.16 | 0.06 | 0.01 |  | * | 0.05 |  |
|  |  | 10 /day | 0.11 | 0.08 | 0.16 |  |  |  |  |
|  |  | Seldom | 0.07 | 0.05 | 0.19 |  |  |  |  |
| cg24843003 (n=124) | **DAZAP1** |  |  |  |  |  |  |  | **↑** |
|  |  | >10 /hr | 0.10 | 0.06 | 0.11 | Gender |  | 0.0007 |  |
|  |  | 1-9 /hr | 0.04 | 0.07 | 0.58 |  |  | 0.01 |  |
|  |  | 10 /day | 0.12 | 0.08 | 0.16 |  |  |  |  |
|  |  | Seldom | 0.14 | 0.06 | 0.01 |  | * |  |  |
| cg15730464 (n=112) | **LGI2** |  |  |  |  |  |  |  | **↓** |
|  |  | >10 /hr | 0.26 | 0.09 | 0.01 | Tobacco Smoke Exposure ( at 10 yrs); SES; Gender | * | 5.6 |  |
|  |  | 1-9 /hr | 0.10 | 0.09 | 0.28 |  |  | 0.02 |  |
|  |  | 10 /day | 0.01 | 0.11 | 0.96 |  |  |  |  |
|  |  | Seldom | 0.17 | 0.08 | 0.04 |  |  |  |  |
| cg07023532 (n=122) | **ACOT4** |  |  |  |  |  |  |  | **↑** |
|  |  | >10 /hr | 0.20 | 0.08 | 0.02 | Gender; BMI | 0 | 2.2 |  |
|  |  | 1-9 /hr | 0.25 | 0.09 | 0.005 |  | * | 0.1 |  |
|  |  | 10 /day | 0.24 | 0.11 | 0.03 |  | 0 |  |  |
|  |  | Seldom | 0.12 | 0.07 | 0.09 |  |  |  |  |
| cg20255272 (n=113) | **VWA1** |  |  |  |  |  |  |  | **↑** |
|  |  | >10 /hr | 0.20 | 0.11 | 0.08 | Tobacco Smoke Exposure (0-4 yrs only); Gender; BMI; SES; |  | 1.9 |  |
|  |  | 1-9 /hr | 0.24 | 0.11 | 0.03 |  |  | 0.2 |  |
|  |  | 10 /day | 0.35 | 0.14 | 0.02 |  | 0 |  |  |
|  |  | Seldom | -0.05 | 0.10 | 0.61 |  |  |  |  |
| cg04154465 (n=115) | **WNT2B** |  |  |  |  |  |  |  | **↑** |
|  |  | >10 /hr | 0.30 | 0.12 | 0.01 | SES; Gender; BMI | * | 9.2 |  |
|  |  | 1-9 /hr | 0.07 | 0.12 | 0.53 |  |  | 0.003 |  |
|  |  | 10 /day | -0.17 | 0.15 | 0.28 |  |  |  |  |
|  |  | Seldom | 0.08 | 0.10 | 0.42 |  |  |  |  |
| cg18459806 (n=106) | **NIN** |  |  |  |  |  |  |  | **↓** |
|  |  | >10 /hr | -0.17 | 0.07 | 0.02 | Maternal Smoking; Tobacco Smoke Exposure (0-4 yrs and at 10 yrs); SES; Gender; BMI; Exposure to smoke outside the home | 0 | 1.6 |  |
|  |  | 1-9 /hr | -0.12 | 0.06 | 0.08 |  |  | 0.2 |  |
|  |  | 10 /day | -0.17 | 0.08 | 0.05 |  |  |  |  |
|  |  | Seldom | -0.12 | 0.06 | 0.04 |  |  |  |  |
| cg17053854 (n=112) | **SEPT9** |  |  |  |  |  |  |  | **↑** |
|  |  | >10 /hr | 0.10 | 0.05 | 0.03 | Tobacco Smoke Exposure (at 10 yrs ); BMI; Gender |  | 10.9 |  |
|  |  | 1-9 /hr | 0.14 | 0.04 | 0.00 |  | ** | 0.001 |  |
|  |  | 10 /day | -0.04 | 0.05 | 0.44 |  |  |  |  |
|  |  | Seldom | 0.02 | 0.04 | 0.55 |  |  |  |  |
| cg25324786 (n=108) | **RASA3** |  |  |  |  |  |  |  | **↑** |
|  |  | >10 /hr | 0.25 | 0.08 | 0.004 | Maternal Smoking; Tobacco Smoke Exposure (0-4 yrs and at 10 yrs); SES; Gender; Exposure to smoke outside the home | * | 12.0 |  |
|  |  | 1-9 /hr | 0.08 | 0.08 | 0.31 |  |  | 0.0008 |  |
|  |  | 10 /day | -0.01 | 0.10 | 0.96 |  |  |  |  |
|  |  | Seldom | -0.01 | 0.07 | 0.92 |  |  |  |  |
| cg05575058 (n=106) | **FAM164A** |  |  |  |  |  |  |  | **↓** |
|  |  | >10 /hr | -0.22 | 0.08 | 0.01 | Maternal Smoking; Tobacco Smoke Exposure (0-4 yrs and at 10 yrs); SES; Gender; BMI; Exposure to smoke outside the home | * | 4.5 |  |
|  |  | 1-9 /hr | -0.10 | 0.08 | 0.19 |  |  | 0.04 |  |
|  |  | 10 /day | -0.11 | 0.10 | 0.27 |  |  |  |  |
|  |  | Seldom | -0.09 | 0.07 | 0.19 |  |  |  |  |
|  |  |  |  |  |  |  |  |  |  |

*** p<0.001

** p<0.01

* p<0.05

◌ p<0.1

Once the Dunnett’ tests provided statistical evidence of differences in marginal means of the heavy vehicular traffic frequency, a second test for trend is performed to assess a ‘dose-response’ relationship.
