# Supplementary material for: Frequency of heavy vehicle traffic and association with DNA methylation at age 18 years in a subset of the Isle of Wight birth cohort
Source: Environ Epigenet. 2019 Jan 23;4(4):dvy028. doi: 10.1093/eep/dvy028 (PMC6343046; doi:10.1093/eep/dvy028)
Supplement: Supplementary Data [file dvy028_supp.zip › ToppGene Results.pdf]

Results

Go To Start Page

Input Parameters [Hide Detail]

Number of genes in training set:	34
Number of genes in test set:	0
Correction and Cutoff:	(see table below)
pValue Method:	Hypergeometric Probability Mass Function
Random sampling size in analysis:	0
Minimun feature count in test set:	2
Analysis took:	0 seconds
Analysis finished at:	Thu Apr 19 15:49:00 EDT 2018

category	Correction	Cutoff	Min	Max
GO: Molecular Function	FDR B&H	0.05	1	2000
GO: Biological Process	FDR B&H	0.05	1	2000
GO: Cellular Component	FDR B&H	0.05	1	2000
Human Phenotype	FDR B&H	0.05	1	2000
Mouse Phenotype	FDR B&H	0.05	1	2000
Domain	FDR B&H	0.05	1	2000
Pathway	FDR B&H	0.05	1	2000
Pubmed	FDR B&H	0.05	1	2000
Interaction	FDR B&H	0.05	1	2000
Cytoband	FDR B&H	0.05	1	2000
Transcription Factor Binding Site	FDR B&H	0.05	1	2000
Gene Family	FDR B&H	0.05	1	2000
Coexpression	FDR B&H	0.05	1	2000
Coexpression Atlas	FDR B&H	0.05	1	2000
Computational	FDR B&H	0.05	1	2000
MicroRNA	FDR B&H	0.05	1	2000
Drug	FDR B&H	0.05	1	2000
Disease	FDR B&H	0.05	1	2000

Training Results [Collapse All] [Download All] [Sparse Matrix] Display pValues and Scores as

Scientific (4 significant digits) ▼ Table row limit 50 ▼

1: GO: Molecular Function [Display Chart] 34 input genes in category / 161 annotations before applied cutoff / 18661 genes in category

	ID	Name	Source	pValue	FDR B&H	FDR B&Y	Bonferroni	Genes from Input	Genes in Annotation
1	GO:0015278	calcium-release channel activity		3.805E-4	3.063E-2	1.734E-1	6.126E-2	2	16
2	GO:0099604	ligand-gated calcium channel activity		3.805E-4	3.063E-2	1.734E-1	6.126E-2	2	16

2: GO: Biological Process [Display Chart] 34 input genes in category / 885 annotations before applied cutoff / 18623 genes in category

No results to display

3: GO: Cellular Component [Display Chart] 34 input genes in category / 146 annotations before applied cutoff / 19061 genes in category

No results to display

4: Human Phenotype [Display Chart] 10 input genes in category / 725 annotations before applied cutoff / 4707 genes in

category

No results to display

5: Mouse Phenotype [Display Chart] 19 input genes in category / 477 annotations before applied cutoff / 9319 genes in category

No results to display

6: Domain [Display Chart] 34 input genes in category / 253 annotations before applied cutoff / 18735 genes in category

	ID	Name	Source	pValue	FDR B&H	FDR B&Y	Bonferroni	Genes from Input	Genes in Annotation
1	PF05483	SCP-1	Pfam	1.818E-3	4.181E-2	2.556E-1	4.600E-1	1	1
2	IPR008496	DUF778	InterPro	1.818E-3	4.181E-2	2.556E-1	4.600E-1	1	1
3	IPR030645	SEPT9	InterPro	1.818E-3	4.181E-2	2.556E-1	4.600E-1	1	1
4	IPR003028	Na/ntran symport glycine GLY1	InterPro	1.818E-3	4.181E-2	2.556E-1	4.600E-1	1	1
5	IPR029664	NIN	InterPro	1.818E-3	4.181E-2	2.556E-1	4.600E-1	1	1

Show 6 more annotations

7: Pathway [Display Chart] 21 input genes in category / 137 annotations before applied cutoff / 12450 genes in category

	ID	Name	Source	pValue	FDR B&H	FDR B&Y	Bonferroni	Genes from Input	Genes in Annotation
1	M3008	Genes encoding structural ECM glycoproteins	MSigDB C2 BIOCARTA (v6.0)	2.890E-4	3.959E-2	2.178E-1	3.959E-2	4	196

8: Pubmed [Display Chart] 34 input genes in category / 1350 annotations before applied cutoff / 36332 genes in category

	ID	Name	Source	pValue	FDR B&H	FDR B&Y	Bonferroni	Genes from Input	Genes in Annotation
1	11555487	Positional cloning of the mouse saccharin preference (Sac) locus.	Pubmed	1.765E-4	3.964E-3	3.086E-2	2.383E-1	2	21
2	24322204	Genome-wide association study of bipolar disorder accounting for effect of body mass index identifies a new risk allele in TCF7L2.	Pubmed	3.164E-4	3.964E-3	3.086E-2	4.272E-1	2	28
3	19851296	Assessment of a polymorphism of SDK1 with hypertension in Japanese Individuals.	Pubmed	3.200E-4	3.964E-3	3.086E-2	4.321E-1	3	142
4	20198315	Association of genetic variants with hemorrhagic stroke in Japanese individuals.	Pubmed	3.267E-4	3.964E-3	3.086E-2	4.410E-1	3	143
5	19724895	Association of gene polymorphisms with chronic kidney disease in Japanese individuals.	Pubmed	6.163E-4	3.964E-3	3.086E-2	8.320E-1	2	39

Show 45 more annotations

9: Interaction [Display Chart] 30 input genes in category / 668 annotations before applied cutoff / 17363 genes in category

No results to display

10: Cytoband [Display Chart] 33 input genes in category / 28 annotations before applied cutoff / 34661 genes in category

	ID	Name	Source	pValue	FDR B&H	FDR B&Y	Bonferroni	Genes from Input	Genes in Annotation
1	1p36.33	1p36.33		1.305E-6	3.655E-5	1.435E-4	3.655E-5	4	85
2	8p23.3	8p23.3		1.332E-4	1.865E-3	7.324E-3	3.730E-3	2	18
3	8q21.12	8q21.12		1.903E-3	1.598E-2	6.276E-2	5.329E-2	1	2
4	8q12-q13	8q12-q13		2.854E-3	1.598E-2	6.276E-2	7.990E-2	1	3
5	1p13-p12	1p13-p12		2.854E-3	1.598E-2	6.276E-2	7.990E-2	1	3

Show 2 more annotations

11: Transcription Factor Binding Site [Display Chart] 16 input genes in category / 200 annotations before applied cutoff /

9770 genes in category

No results to display

12: Gene Family [Display Chart] 26 input genes in category / 29 annotations before applied cutoff / 18194 genes in category

	ID	Name	Source	pValue	FDR B&H	FDR B&Y	Bonferroni	Genes from Input	Genes in Annotation
1	682	Zinc fingers FYVE-type|Pleckstrin homology domain containing|Rho guanine nucleotide exchange factors	genenames.org	3.069E-3	4.861E-2	1.926E-1	8.901E-2	3	206
2	677	Peptidyl arginine deiminases	genenames.org	7.126E-3	4.861E-2	1.926E-1	2.066E-1	1	5
3	1185	CD molecules|Type I classical cadherins	genenames.org	7.126E-3	4.861E-2	1.926E-1	2.066E-1	1	5
4	658	Fibronectin type III domain containing|I-set domain containing|Myosin binding proteins	genenames.org	1.138E-2	4.861E-2	1.926E-1	3.299E-1	1	8
5	1230	WD repeat domain containing|BEACH domain containing	genenames.org	1.279E-2	4.861E-2	1.926E-1	3.709E-1	1	9

Show 11 more annotations

13: Coexpression [Display Chart] 34 input genes in category / 2244 annotations before applied cutoff / 23137 genes in category

	ID	Name	Source	pValue	FDR B&H	FDR B&Y	Bonferroni	Genes from Input	Genes in Annotation
1	17414055-Table2	Human pancreas Kim07 34genes	GeneSigDB	1.267E-5	2.843E-2	2.358E-1	2.843E-2	3	31
2	M4391	Genes up-regulated in the HMEC cells (primary mammary epithelium) upon expression of TP53 [GeneID=7157] off adenoviral vector.	MSigDB C2: CGP Curated Gene Sets (v6.0)	3.537E-5	3.969E-2	3.292E-1	7.938E-2	9	1172

14: Coexpression Atlas [Display Chart] 33 input genes in category / 1642 annotations before applied cutoff / 23956 genes in category

No results to display

15: Computational [Display Chart] 16 input genes in category / 86 annotations before applied cutoff / 10037 genes in category

No results to display

16: MicroRNA [Display Chart] 34 input genes in category / 1945 annotations before applied cutoff / 19844 genes in category

No results to display

17: Drug [Display Chart] 34 input genes in category / 4571 annotations before applied cutoff / 22841 genes in category

	ID	Name	Source	pValue	FDR B&H	FDR B&Y	Bonferroni	Genes from Input	Genes in Annotation
1	5667 UP	Metyrapone [54-36-4]; Up 200; 17.6uM; MCF7; HT HG-U133A	Broad Institute CMAP Up	9.093E-6	2.320E-2	2.089E-1	4.157E-2	5	192
2	ctd:D019287	Zinc Sulfate	CTD	1.015E-5	2.320E-2	2.089E-1	4.639E-2	8	741

18: Disease [Display Chart] 27 input genes in category / 342 annotations before applied cutoff / 16205 genes in category

	ID	Name	Source	pValue	FDR B&H	FDR B&Y	Bonferroni	Genes from Input	Genes in Annotation
1	C0424711	Orbital separation diminished	DisGeNET Curated	6.619E-4	4.077E-2	2.615E-1	2.264E-1	2	23
2	cv:CN158704	Seckel syndrome 7	Clinical Variations	1.669E-3	4.077E-2	2.615E-1	5.707E-1	1	1
3	C0563305	IgA myeloma	DisGeNET BeFree	1.669E-3	4.077E-2	2.615E-1	5.707E-1	1	1
4	C1446219	Akinetic rigid syndrome	DisGeNET BeFree	1.669E-3	4.077E-2	2.615E-1	5.707E-1	1	1

5	OMIN:162100	AMYOTROPHY, HEREDITARY NEURALGIC; HNA	OMIM	1.669E-3	4.077E-2	2.615E-1	5.707E-1	1	1

Show 18 more annotations
